# Anomalous metal segregation in lithium-rich material provides design rules for stable cathode in lithium-ion battery

**DOI:** 10.1038/s41467-019-09248-0

**Published:** 2019-04-09

**Authors:** Ruoqian Lin, Enyuan Hu, Mingjie Liu, Yi Wang, Hao Cheng, Jinpeng Wu, Jin-Cheng Zheng, Qin Wu, Seongmin Bak, Xiao Tong, Rui Zhang, Wanli Yang, Kristin A. Persson, Xiqian Yu, Xiao-Qing Yang, Huolin L. Xin

**Affiliations:** 1 Center for Functional Nanomaterials, Brookhaven National Laboratory, Upton, NY 11973 USA; 2 Chemistry Division, Brookhaven National Laboratory, Upton, NY 11973 USA; 3 Beijing Advanced Innovation Center for Materials Genome Engineering, Institute of Physics, Chinese Academy of Sciences, 100190 Beijing, China; 4 Department of Physics and the Collaborative Innovation Center for Optoelectronic Semiconductors and Efficient Devices, Xiamen University, 361005 Xiamen, China; 5 Advanced Light Source, Lawrence Berkeley National Laboratory, 1 Cyclotron Road, Berkeley, CA 94720 USA; 6 Xiamen University Malaysia, 439000 Sepang, Selangor Malaysia; 7 Department of Physics and Astronomy, University of California, Irvine, CA 92697 USA; 8 Energy Storage and Distributed Resources Division, Lawrence Berkeley National Laboratory, Berkeley, CA 94720 USA; 9 Department of Materials Science, University of California Berkeley, Berkeley, CA 94720 USA; 10 Present Address: Department of Physics and Astronomy, University of California, Irvine, CA 92697 USA

## Abstract

Despite the importance of studying the instability of delithiated cathode materials, it remains difficult to underpin the degradation mechanism of lithium-rich cathode materials due to the complication of combined chemical and structural evolutions. Herein, we use state-of-the-art electron microscopy tools, in conjunction with synchrotron X-ray techniques and first-principle calculations to study a 4*d*-element-containing compound, Li_2_Ru_0.5_Mn_0.5_O_3_. We find surprisingly, after cycling, ruthenium segregates out as metallic nanoclusters on the reconstructed surface. Our calculations show that the unexpected ruthenium metal segregation is due to its thermodynamic insolubility in the oxygen deprived surface. This insolubility can disrupt the reconstructed surface, which explains the formation of a porous structure in this material. This work reveals the importance of studying the thermodynamic stability of the reconstructed film on the cathode materials and offers a theoretical guidance for choosing manganese substituting elements in lithium-rich as well as stoichiometric layer-layer compounds for stabilizing the cathode surface.

## Abstract (web summary)

The authors show that after cycling the model cathode material Li_2_Ru_0.5_Mn_0.5_O_3_ undergoes ruthenium segregation and even exsolution at the reconstructed oxide surface, which triggers a progressive degradation process. The insights enable new dimensions in choosing dopants for stabilized cathode surface.

## Introduction

Designing layered cathode materials with high reversible lithium storage capacity, as well as structural and chemical stabilities, is essential for developing new generations of lithium-ion batteries with long cycle life. Lithium-rich layered oxides, in particular, are a class of high-capacity layered cathode materials that can meet the high energy density demand of battery electric vehicles^1–3^. These oxides, however, are prone to significant oxygen loss during charge/discharge cycles, which in turn leads to notable voltage and capacity fading^4–8^. The cathode degradation problem can be improved by surface treatment or introducing a content concentration gradient^9,10^. Metal oxide such as ZnO, MgO, Al_2_O_3_^11–13^, metal fluoride such as AlF_3_, or metal phosphastes such as AlPO_4_ or CoPO_4_ have been widely used as a surface coating layer to protect the cathode materials from the attack of HF, which is decomposed from electrolyt^14–17^. However, poor electronic conductivity and surface roughness are two of the main problems of current surface coating technologies, which can be greatly improved by introducing a concentration gradient or a core shell structure at the secondary particle level. Cation doping with electrochemical inactive elements, such as Al, Ti, Mg, has also been widely used to enhance the crystal structure stability of lithium-rich layered cathode materials^18–21^, and a recent computational study shows that Os, Sb, Ru, Ir, or Ta are the top-ranking dopants that can retain oxygens on the surface of Li_2_MnO_3_^22^. All these indicate that the chemical and structural stability of the surface could be one of the key contributors to the enhanced cathode performance^10,23–27^. Apart from surface treatment, at the bulk level, there has been a new strategy of using 4*d* or 5*d* transition metals to stabilize the crystal structure against oxygen release during the high voltage charging process^28,29^. Specifically, a series of new model compounds, Li_2_IrO_3_ for example^30^, has been successfully applied to investigating the oxygen anion redox contribution to the charge capacity. However, unlike their 3*d* counterparts, most prior studies of 4*d* or 5*d* element containing layered oxides have focused on the pristine state of these compounds but overlooked the cycling-induced oxygen loss and surface reconstruction. Therefore, it is desirable to study how instabilities of the surface-reconstructed layer can lead to different bulk degradation pathways in the 4*d* or 5*d* element containing layered oxide systems. It is of great importance to utilize multi-scale mechanistic characterization tools that can make a clear connection between the degradation pathway of the bulk and the surface properties.

Here we report the investigation results of a high-capacity lithium-rich 3*d*-4*d* transition-metal-layered compound, namely Li_2_Ru_0.5_Mn_0.5_O_3_ or lithium-rich ruthenium-manganese oxide (LRMO). Replacing manganese by ruthenium in a lithium-rich structure, Li_2_MnO_3_, has many benefits, such as increasing the electronic conductivity and thermal stability against the oxygen release during heating, as long as the delithiation level is not too high. Therefore, the incorporation of 4*d* transition metal, Ru here, provides a valuable model compound to study the effects of 4*d* metals on the structural and chemical stability^6,28,31,32^, as well as their compatibility with 3*d* transition metals (TM). Specifically, the model compound, LRMO, can reach a high capacity of 300 mAh g^−1^, but still suffers from the voltage and capacity fading during high voltage electrochemical cycling^31^, which has similarity to its 3*d* counterpart, i.e., lithium- and manganese-rich nickel-manganese cobalt oxide (LMR-NMC)^33–35^. Using this compound, we provide a scrutiny of what would happen when severe oxygen loss occurs in cells that are charged to high voltages.

We find that after extended cycling, a three-dimensional porous structure is formed in LRMO and segregation of ruthenium and manganese at the submicron scale can be observed. More interestingly and surprisingly, we find that ruthenium is expelled from the reconstructed oxide surface and forms metallic clusters at the nanoscale. In conjunction with ab initio calculations, this study reveals the intricate connection between the instability of the reconstructed layer at the surface and the degradation of the layered cathode materials in bulk. At the first glance, the metallic Ru segregation effect seems to contradict Shin et al.’s computational prediction that Ru is a high-ranking oxygen retention dopant on the surface of Li_2_MnO_3_^22^. On the contrary, the two studies investigate surface stability of lithium-rick oxides in different regimes of oxygen release. In this study the consideration of the surface reconstruction phase, when severe oxygen release takes place in deep charging states, adds an additional constraint to guide the screening of surface dopants. Our DFT calculations identify that 3*d* elements, Sc, Ti, V, Cr, and 4*d* elements, Y, Zr, Nb, Mo can be added to the surface to conceal capacity contributing but less stable elements in the bulk. Based on the theoretical calculation, we synthesize a lithium-rich nickel-titanium-niobium oxide material, expecting that titanium and niobium can stabilize the surface. Imaging results of this material show no hint of elemental segregation even after 50 charge/discharge cycles, which experimentally validates our theoretical prediction.

## Results

### Electrochemical measurement

**Fig. 1:**
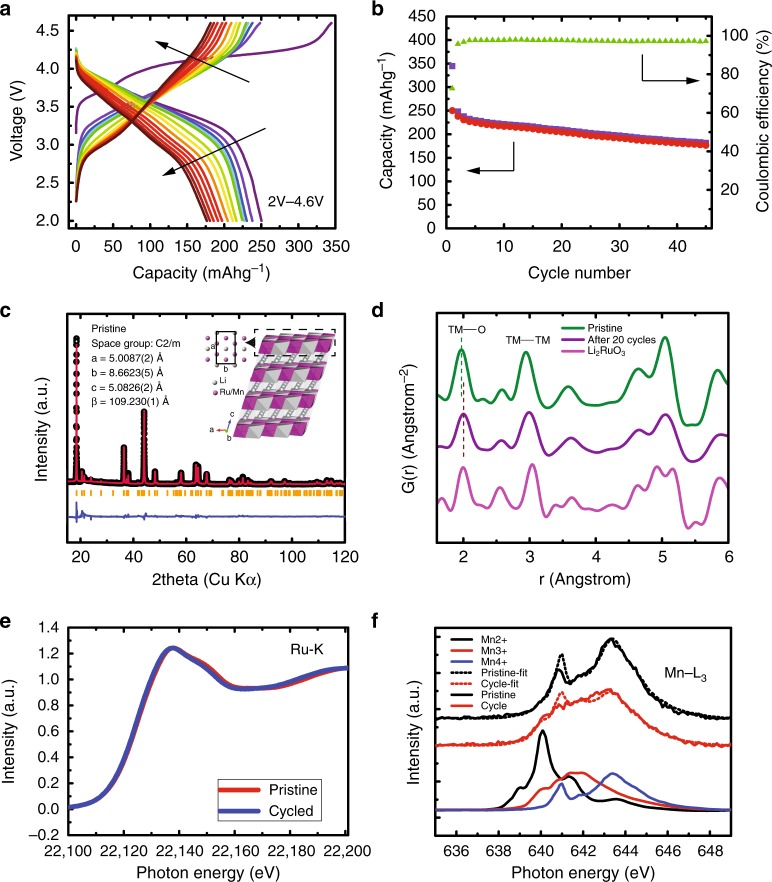
Electrochemical, X-ray pair distribution function and absorption analysis of Li_2_Ru_0.5_Mn_0.5_O_3_ (LRMO). **a** Charge/discharge curves of LRMO/lithium half-cell with cutoff voltages of 2 V and 4.7 V. **b** Charge/discharge capacity and coulombic efficiency of LRMO half-cell as a function of charge cycle. **c** Synchrotron X-ray powder diffraction measurement of pristine LRMO (inset: atomic model of LRMO). **d** X-ray pair distribution function of pristine LRMO, LRMO after 20 cycles, and Li_2_RuO_3_ as reference samples. **e** Hard X-ray absorption spectra of the pristine LRMO and LRMO after 15 cycles for the Ru K edge. **f** Soft X-ray absorption spectra of the pristine LRMO and LRMO after 15 cycles for the Mn L_3_ edge

The electrochemical performance of LRMO materials was tested using lithium half-cells in standard 2032 coin-cells. The cycled electrodes were collected for the subsequent chemical sensitive electron tomography, aberration-corrected electron microscopy, and X-ray spectroscopy, as well as diffraction studies. The half-cell was cycled between 2.0 V and 4.6 V. Figure 1a shows the electrochemistry behavior of LRMO material at a charge/discharge rate of C/10 (see the method section for details). It shows a high voltage plateau at around 4.2 V in the first charging process and a large first-charge capacity of 290 mAh/g. It has been widely accepted that the high voltage plateau is associated with the anionic redox activation^32,36–39^. With continued cycling, capacity and voltage fading were observed for this high voltage charge limit cycling (Fig. 1a, b).

### Bulk structure and chemistry

The lithium-rich layered structure proposed for LRMO shown in the inset of Fig. 1c is verified by an X-ray powder diffraction measurement (Fig. 1). Because pair distribution function (PDF) measurement is sensitive to both short-range and long-range ordering of structures, we investigated the ensemble structural change of the oxide lattice by X-ray pair distribution function (PDF) measurement of a pristine electrode and an electrode after the twentieth cycle (Fig. 1d and Supplementary Fig. S15). By comparing with the PDF of a Li_2_RuO_3_ reference, it shows that the pristine LRMO material retains the structure of Li_2_RuO_3_ with reduced lattice parameter values due to the inclusion of the smaller manganese ions. After 20 cycles, however, all peaks in the LRMO PDF become weaker and broader (also see Supplementary Fig. 15). This is indicative of the increased disorder in the structure, which suggests that the long-range ordered oxide lattice has been broken into smaller domains^40^. This finding agrees with our electron microscopy results that will be shown next. In addition, the transition metal-oxygen (TM-O) peak shifts to a larger distance, indicating the reduction of transition metal and the loss of oxygen^41^. Apart from structural measurement, hard and soft X-ray absorption spectroscopy are employed to study the change of local bonding environment of Ru and Mn. Figure 1e shows the X-ray absorption near-edge structure (XANES) of Ru K edge before and after cycling. The change in the XANES spectra, although small, is noticeable showing that there is a very slight reduction Ru at the bulk level; however, the change of Ru oxidation state is too little to be quantified with statistical significance. On the other hand, soft X-ray absorption spectra of Mn L_3_ edge measured in the partial-fluorescence-yield (PFY) mode and the total-electron-yield (TEY) mode show a more noticeable reduction of the valence state. By fitting the PFY Mn L_3_ edge with a linear combination of Mn^2+,3+,4+^ reference spectra, Mn’s bonding environments are decomposed as Mn^2+^:Mn^3+^:Mn^4+^ = 0.024:0.138:0.838 for the pristine material and Mn^2+^:Mn^3+^:Mn^4+^ = 0.039:0.402:0.559 after 15 cycles. The considerable reduction of Mn suggest that oxygen loss likely occurred. The decomposition of TEY Mn L_3_ edge shows more prominent valence reduction, i.e., Mn^2+^:Mn^3+^:Mn^4+^ = 0.220:0.441:0.339 after 10 cycles and Mn^2+^:Mn^3+^:Mn^4+^ = 0.320:0.517:0.163 after 17 cycles (It is worth noting that s-XAS has a probe depth of ~100 nm in the PFY mode and a probe depth of ~10 nm in the TEY mode. Therefore the TEY spectral information reflects Mn reduction on the surface, which typically is more degraded than the bulk; whereas, the Mn valence quantification of the PFY spectra here is considered qualitative for the bulk information). All these synchrotron X-ray measurements suggest although the overall oxide structure is maintained, irreversible crystal and microstructural degradation occurred in the LRMO materials during cycling.

### Morphological and chemical change at the particle and the atomic scales

**Fig. 2:**
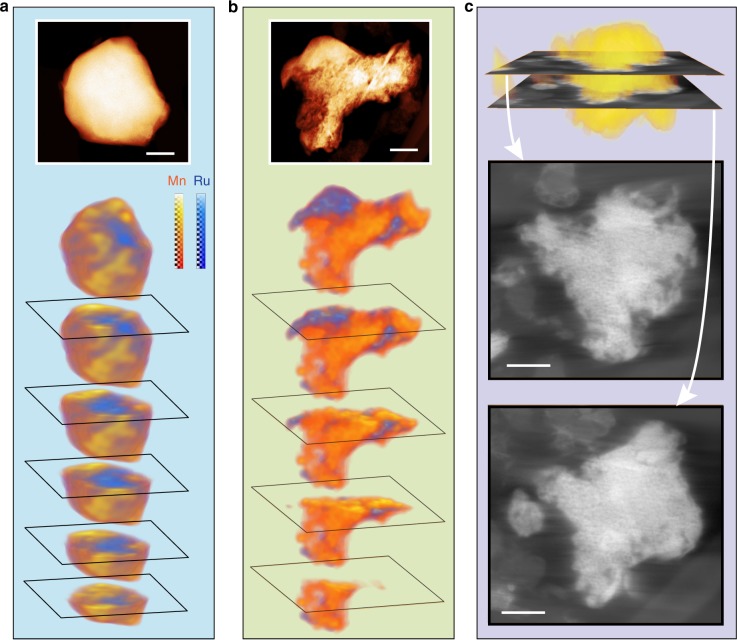
Three-dimensional visualization of the chemical and structural change of Li_2_Ru_0.5_Mn_0.5_O_3_ (LRMO) before and after extended charge/discharge cycling. Three-dimensional distribution of Ru and Mn of **a** pristine LRMO and **b** LRMO after 97 charge/discharge cycles reconstructed by STEM-EDX tomography. **c** annular dark-field STEM tomographic (ADF-STEM) reconstruction of a LRMO primary particle after 97 cycles. Two cross-sectional images of the three-dimensional reconstruction are presented to demonstrate that a porous structure had developed throughout the particle (also see Supplementary Figs. 2 and 16 and Supplementary Movie 2). (Scale bar: 100 nm)

To obtain a direct visualization of the structural changes, electron tomography and atomic-resolution transmission electron microscopy study were performed. Using energy dispersive X-ray spectroscopic (EDX) mapping in the scanning transmission electron microscopy (STEM) mode, in conjunction with electron tomography, we obtained the chemical distribution of ruthenium and manganese in the LRMO primary particles in three dimensions. Figure 2a shows that the as-prepared sample has a manganese-rich surface and a ruthenium-rich core (also see Supplementary Movie 1). Apart from some minor content inhomogeneity, the as-prepared primary particles are generally in uniform solid solution structure without observable internal defects or pores (see Supplementary Figures 1 and 21 and Movie 2 for the visualization of the internal structures of a pristine particle). It is worth noting that the LRMO primary particles are terminated with rounded surface with sparseness of well-defined facets (Supplementary Figs. 1, 23, and 26). This “potato-like” crystal habit is different from these of the materials made using co-precipitation or molten-salt methods, which typical provide primary particles with well-defined facets^35^. After 97 charge/discharge cycles, however, the chemical distribution of manganese and ruthenium in a fraction of the particles was significantly different from the pristine sample as shown in the reconstructed STEM-EDX tomography images in Fig. 2b and Supplementary Movie 3. A significant chemical segregation is observed at the scale of tens of nanometers. To obtain a higher resolution view of the internal structural changes of the primary particles, high-resolution tomography tilt series using annular dark-field scanning transmission electron microscopy (ADF-STEM) was collected and the reconstructed 3D result is shown in Fig. 2c, Supplementary Figure 2, and Movie 4. They clearly show that the interior of the primary particle has been significantly degraded: a porous structure throughout the entire particle is formed (also see Supplementary Fig. 16 a single projection taken at moderate dose condition shows the porous structure). This is in sharp contrast to the pristine sample where a solid and uniform internal structure was observed (Fig. 2a, Supplementary Fig. 1, and Mov. 2).

**Fig. 3:**
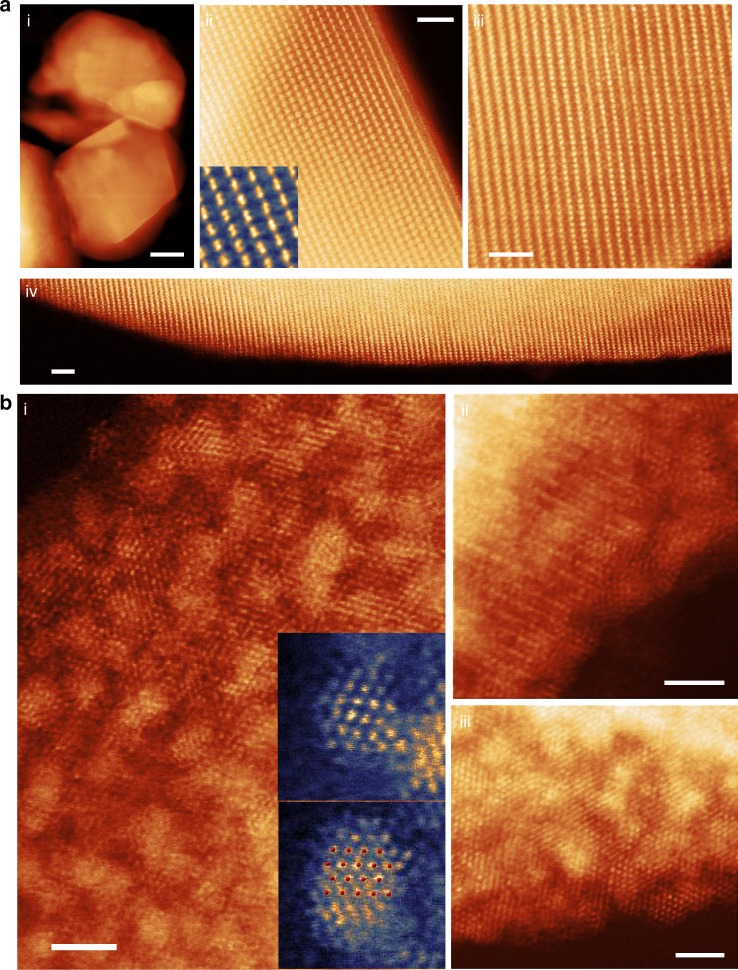
Atomic-scale imaging of the near-surface structure of primary particles. Aberration-corrected high-angle annular dark-field scanning transmission electron microscopy (Z-contrast STEM) image of **a** pristine LRMO and **b** LRMO after 15 cycles. **a** Atomic-resolution Z-contrast images show dark lithium diffusion channels extend from the interior of the particle to the surface indicating that the structure at the surface is nearly the same as the bulk. **b** Images show the near-surface area of LRMO was significantly disrupted after 15 charge/discharge cycles. High-intensity clusters are observed over the entire surface. Insets of **b** (i) show atomic-resolution images of the segregated clusters agree with the projected structure of metallic ruthenium (atomic structure overlaid in red). (Scale bar in **a** (i) 100 nm; scale bar in the rest: 2 nm)

To investigate the atomic structure evolution, an extensive number of aberration-corrected high-angle annular dark-field scanning transmission electron microscopy (HAADF-STEM) images were recorded. Only some of the representative ones are shown here in Fig. 3 and Supplementary Figures 20, 26 and 27. Because HAADF-STEM uses high-angle scattered electrons to form images, the contrast of the resulting image is sensitive to the projected atomic mass of the underlying atomic columns. This contrast is commonly referred to as Z-contrast^42^. Therefore, when the layered oxide is oriented such that the *c*-axis basal planes are parallel to the electron beam, the atomic-resolution Z-contrast image would show an alternation of transition metal (higher intensity) and lithium (lower intensity) layers with a period of ~4.8 angstroms. Figure 3a(ii, iii, iv) and Supplementary Figures 20, 26 and 27 show that the projected structure of the pristine oxide follows this layered pattern, and the lattice maintains coherence from the surface to the interior/bulk as expected from previous PDF results. It is worth noting that in the pristine sample, all lithium channels remain dark with nearly no observable surface reconstruction layer. To support this claim, a wide field image of the surface is shown in Fig. 3a(iv) and images of other surfaces/facets are shown in Supplementary Figures 20, 26, 27. However, upon cycling, drastic structural changes occurred in the material as shown in Fig. 3b and Supplementary Figure 3. The oxide backbones are significantly disrupted and small 1–2 nm clusters are segregated out onto the surfaces. In addition, based on the Z-contrast STEM image, the near-surface portion of the lithium channels are filled with transition metals (Fig. 3b(ii) and for details see Supplementary Fig. 4). Furthermore, we analyzed the segregated nanoparticles on the surface. The higher intensity of the segregated nanoparticles on the surface indicates that they likely contain ruthenium atoms. This is further confirmed by STEM-EDX mapping showing that the high-intensity particles are clearly rich in ruthenium (Supplementary Fig. 5). Interestingly, we found that the atomic structure of the segregated nanoparticles agrees well with the hexagonal close packed ruthenium metal, as shown in the inset picture in Fig. 3b. Such metallic segregation phenomenon at the atomic scale has never been reported before in lithium-ion battery cathode materials. To eliminate the possibility that the formation of clusters is caused by a reaction that happens at the lower voltage region during electrochemical cycling, we raised the lower cutoff voltage to 3.5 V. Supplementary Figure 6 shows that in this condition, after one charge/discharge cycle, high-intensity clusters are still observed in the near-surface region of the LRMO particles, confirming that the formation of these clusters is not driven by an electrochemical conversion reaction.

### Surface chemistry

**Fig. 4:**
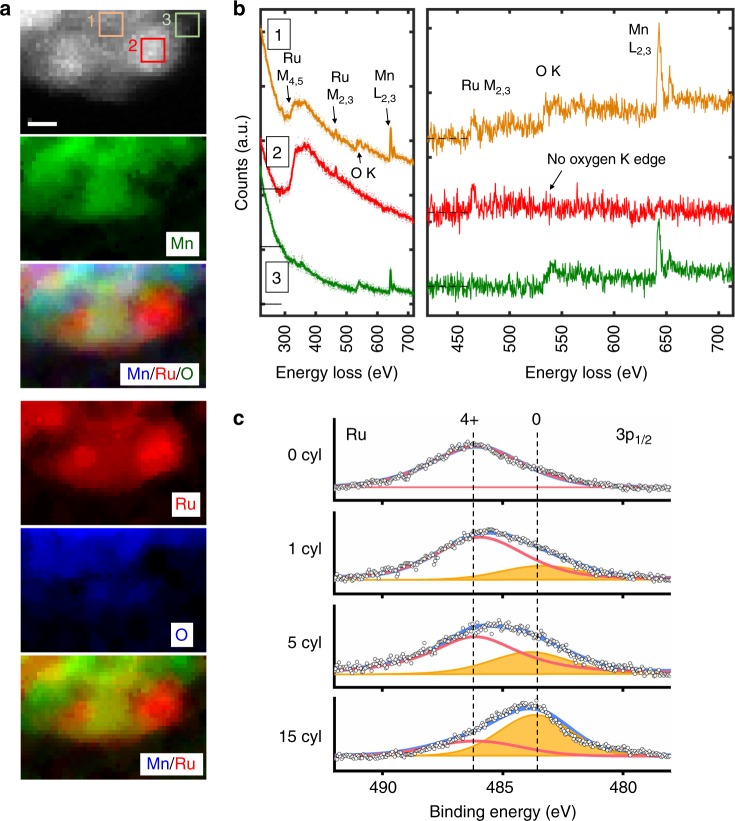
Revealing the surface chemistry by electron energy loss spectroscopy (EELS) and X-ray photoemission spectroscopy (XPS). **a** STEM-EELS mapping of the Mn, Ru, and O in the near-surface area of a LRMO particle after 15 charge cycles. The Ru and O maps show that cluster on the right is nearly pure Ru without oxygen. **b** The EELS spectra integrated from area 1, 2, and 3 in **a**. **c** The Ru 3p_1/2_ core-level XPS spectra of the LRMO cathode surface as a function of charge/discharge cycles. The Ru^4+^ composition decreases and the Ru^0^ composition increases as the cathode undergoes more cycles. (Scale bar: 1 nm)

To confirm the segregated particles are ruthenium metal clusters, we further investigated the chemistry of the segregated particles using spatially-resolved electron energy loss spectroscopy (STEM-EELS) and X-ray photoelectron spectroscopy (XPS) with around 5 nm probe depth. The STEM-EELS chemical maps, shown in Fig. 4, indicate three different regions: the first region has all three elements, Ru, Mn, and O (marked as region 1 in Fig. 4a); the second region has Ru only (marked as region 2); and the third region has Mn and O and little Ru (marked as region 3). Figure 4b shows the spectra integrated from the three marked areas in Fig. 4a. It is interesting to note that in region 2, both ruthenium *M*_4,5_ and *M*_3,4_ edges are prominent, but no signal representing oxygen *K*-edge and Mn *L*_2,3_ can be detected. In region 3, both manganese *L*_2,3_ and oxygen *K* edges are present with very little observable signal representing ruthenium *M*_4,5_ and *M*_3,4_ edges. These results show that the ruthenium is segregated from manganese oxide framework and fully reduced to a metallic state.

To understand the chemical environment of Mn and its spatial variance, we performed a near-edge fine structure mapping of the O-*K* edge and the Mn *L*_2,3_ edges in the near-surface region of a cycled primary particle (Supplementary Fig. 7). The results show that there is a clear down-shift of the Mn *L*_3_ white line and a reduction of *L*_3_/*L*_2_ ratio as the probed area moves closer to the surface, which indicate that the valence of Mn decreases when approaching oxide surface^43,44^. At the very surface, the Mn spectral shape has a close reproduction of the near-edge fine structures of Mn^2+^, which is consistent with our XPS results (Supplementary Fig. 24); whereas the area that is 6 nm away from the surface, a Mn^3+^ fingerprint and *L*_*3*_*/L*_*2*_ ratio are shown, which is in agreement with the soft-XAS TEY results (Supplementary Fig. 7e). The pre-peak of the O-*K* edge, that probes the unfilled O 2*p*-TM 3*d* hybridized states, shows the same trend; the pre-peak intensity is reduced close to the surface area, which indicates a reduction of the transition metals in the oxide. The Mn^2+^ surface layer becomes much thicker in the electrode after the 120 cycles (Supplementary Fig. 25). All these results suggest that there is a Mn-O rock-salt or a Li_1-x_Mn_1+x_O_2_ lithium-containing disordered rock-salt reconstruction layer formed on the surface due to oxygen loss.

To be cautious about the potential artifacts induced by radiation damage of the electron beam, the EELS map was collected with limited dosage. To fully eliminate this concern, we also performed X-ray photoelectron spectroscopy (XPS) study on the cathode surface to confirm the EELS results, since the radiation damage caused by a broad X-ray beam in this type of material is negligible. The XPS results of ruthenium 3*p*_1/2_ and 2*p*_3/2_ core-level spectroscopy, plotted in Fig. 4c and Supplementary Figure S22, clearly show that the reduction of ruthenium from Ru^4+^ to Ru^0^ took place on the surface through cycling. This does not only confirm our atomic-resolution imaging and EELS results, but also indicates that the reduction of Ru^4+^ and formation of metallic Ru, occurred not only in limited regions, but also over most parts of the LRMO cathode surfaces, since XPS probe a large area of the sample surface. The XPS spectra of Mn 2*p*, as shown in Supplementary Figure 24, shows that after 120 charge/discharge cycles, manganese is reduced from 4+ to 2+ valence state, which is in agreement with our STEM-EELS results shown in Supplementary Figure 25.

### Theoretical understanding and prediction

The formation of metallic ruthenium on the surface of the cathode is unexpected and surprising, but it aligns with the observed segregation of ruthenium and manganese at the submicron-scale by the chemical sensitive tomography technique. Our PDF results suggest that the layered oxide backbone in the bulk is still preserved after charge/discharge cycling, which is in good agreement with our atomic-resolution images. However, the STEM-EELS and XPS results suggest that a rock-salt/lithium-containing disordered rock-salt surface reconstruction occurred on the cathode surface. Since ruthenium and manganese are intermixable in the pristine lithium-rich layered oxide structure, we highly suspect that the segregation of metallic ruthenium is due to its incompatibility with the surface-reconstructed rock-salt/lithium-containing disordered rock-salt environment.

**Fig. 5:**
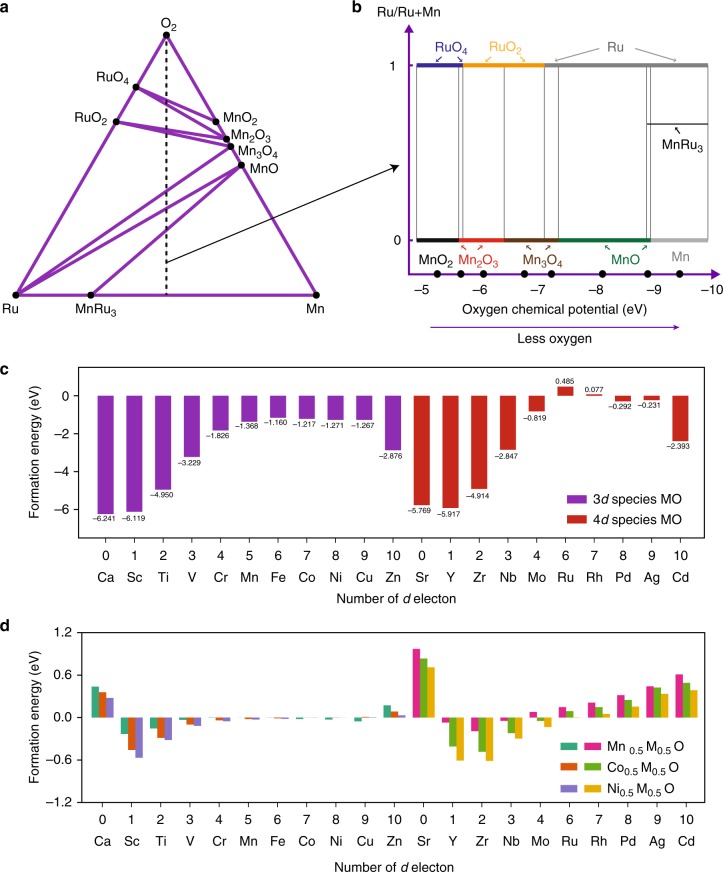
Ab initio calculation for ruthenium segregation and prediction for Mn/Co/Ni substitution that can stabilize the surface. **a** Ternary phase diagram showing the complete convex hull for the system. **b** Presentation of stable phases as a function of oxygen chemical potential phases along the bisecting line of the phase diagram. **c** Ab initio DFT calculation of the formation energy of metal-oxygen (MO) rock-salt structure. **d** Ab initio DFT calculation of the solubility of 3*d*/4*d* transition metals in MnO, NiO, and CoO. (See Supplementary Figure 9 for the full solubility calculation)

To explore this hypothesis, we performed a full convex-hull calculation for the Ru-Mn-O system. It is worthwhile to mention that transition metal segregation and surface reconstruction, from layered structure to spinel structure and finally to rock-salt/lithium-containing disordered rock-salt structure, are gradual degradation processes upon electrochemical cycling. Figure 5a shows the calculated ternary phase diagram and Fig. 5b presents the stable phases along the bisecting line of the phase diagram for Ru:Mn = 1:1 with changing oxygen chemical potential. It shows that under equilibrium conditions, for the full range of manganese being 2+ valence state, ruthenium is only stable in its metallic form. It means it is thermodynamically favorable for ruthenium to reduce to metallic state, which then subsequently segregate out as pure ruthenium clusters in the degraded rock-salt environment.

Our full convex-hull calculation explains why ruthenium spontaneously segregate out on the surface of LRMO and form metal clusters. More importantly, it illustrates that even if ruthenium and manganese are intermixable in their respective parent layered compounds, oxygen loss and surface reconstruction can induce reduction and subsequent segregation/dealloying effects. In comparison, in 3*d* layered oxides, such as LMR-NMC, the loss of oxygen and collapse of the lithium channels similarly occurs at the surface. The surface reconstruction layer is typically an NMC spinel/rock-salt oxide. Even though it impedes lithium diffusion, it acts as a relatively stable passivation film because NiO, MnO, and CoO are intermixable at any ratios^26,45^. However, in our LRMO case, the reconstructed surface is thermodynamically unstable and can spontaneously decompose at room temperature (see Supplementary Fig. 8 for ab initio MD calculation revealing the fast kinetics of this decomposition reaction at room temperature.). It is disrupted into patches due to the segregation of ruthenium metal clusters. This renders the reconstructed layer ineffective in passivating the cathode surface because the disruption exposes new LRMO surface. This process may repeat itself until the LRMO bulk is fully degraded and hence such a recursive surface disruption directly impacts the bulk structure (see Supplementary Fig. 17). In our LRMO case, the porous morphology is likely a direct consequence of the recursive disruption pathway—i.e., the domino effect or chain reaction—and such phenomenon has also been observed in LRMO at the secondary particle level^46^.

By combining experiments and ab initio calculations, we have studied the relationship between the stability of the surface reconstruction film and the bulk degradation pathway. When choosing transition metal cations for the surface of layered cathode, rather than only looking at the stability of the layered oxides in their pristine structure, it is equally important to look at the stability and intermixibility of the elements in the reconstructed metal-oxygen (MO) rock-salt/lithium-containing Li_1-*x*_Mn_1+*x*_O_2_ disordered rock-salt phase. To guide future synthesis, we calculated the formation energy of MO for 3*d* and 4*d* transition elements and their intersolubility with MnO, NiO, and CoO because Mn, Ni, Co are the most widely used elements that can form/stabilize stoichiometric and lithium-rich layered oxide. The trend in Fig. 5c is very clear such that Ru and Rh exhibit positive formation energies and hence possess a thermodynamic driving force for segregation. By further requiring an intermixability with Mn, Ni, and Co, our calculation can narrow the selection to a handful candidates (Fig. 5d and Supplementary Fig. 9), specifically 3*d* elements, Sc, Ti, V, Cr, and 4*d* elements, Y, Zr, Nb, Mo (Molybdenum is a borderline candidate, which mix well with Co and Ni but not Mn. It is also worth noting that even though the CaO are SrO are stable in MO, they are not intermixable with MnO/NiO/CoO). These elements can be added to the surface to conceal other active elements, Ru for example, in the bulk. We prospect that only a few atomic layers of doping with these recommended elements can greatly improve the surface stability of the material without sacrificing the energy density and rate capability.

## Discussion

Based on our aforementioned theoretical calculations, we synthesized a lithium-rich nickel-titanium-niobium oxide material, expecting that titanium and niobium can stabilize the surface (see supplementary methods for details). Additional, niobium is chosen for it higher intensity in Z-contrast imaging—any segregation of niobium can be easily observed in Z-contrast STEM without ambiguity. The electrochemical performance of lithium-rich nickel-titanium-niobium oxide material is shown in Supplementary Figure 10. This material was imaged in its pristine state, after 15, and 50 cycles, respectively (Supplementary Figs. 11, 12, and 13). The atomic-resolution images of the near-surface area of the oxide material show no hint of elemental segregation even after 50 charge/discharge cycles. This result experimentally validates our theoretical calculation.

Here, we only consider using lithium nickel-manganese-cobalt oxide or Li_2_MnO_3_ as the backbone structure as these are widely studied by the lithium-ion battery community for their potential application in next generation batteries for electric vehicles. For other cathode chemistry, for example Sn-, Ti-, Mo-, Nb-based lithium-rich oxide, additional intermixibility diagrams need to be calculated. Additionally, we want to point out that designing a cathode material requires much more consideration than reconstructed surface stability, and our study provides new concepts and an additional design rule for increasing the long-term cyclability of layered oxides. Of course, surface chemistry and uniformity are only two of the important factors for novel lithium-rich cathode material surface coating design. Many other factors, including electronic/ionic conductivity, energy density, side reaction with electrolyte, and manufactural cost should also be taken into consideration.

Additionally, we would like to point out that the ruthenium-manganese system has high thermal stability as long as the delithiation level is not too high. This is also indicative that the Ru segregation, metallic Ru formation, and the resulting porous morphology could be avoided if the battery cell is not charged to high voltage or the surface of the material is well protected (e.g., concentration gradient, surface coating, electrolyte additives). On the other hand, in this paper, we provide an interesting study to show what would happen when oxygen loss occurs in cells that are deep charged or overheated.

A multi-dimensional study on the anomalous structural and chemical evolution in Li_2_Ru_0.5_Mn_0.5_O_3_ is presented in this paper, which combines synchrotron-based X-ray techniques, atomic-scale imaging and spectroscopy, and three-dimensional tomography. Our observations indicate that this oxide undergoes a chemical segregation/dealloying process triggered by high level of delithiation in the bulk and the oxygen loss at the surface. More importantly, the metallic ruthenium clusters are formed and are randomly distributed on the surfaces, which may in turn facilitate a recursive degradation process similar to the domino effect. Our findings provide a new and experimentally validated concept in guiding the selections of proper transition metal elements for the surface of cathode materials in lithium-ion batteries.

## Methods

### Synthesis of lilthium-rich ruthenium-manganese oxide

The Li_2_Ru_0.5_Mn_0.5_O_3_ samples were prepared by a solid state reaction method. Li_2_CO_3_ (Alfa Aesar, 99%), MnCO_3_ (Alfa Aesar, 99.9%), and RuO_2_ (Alfa Aesar, 99.9%) (5 wt. % excess of Li_2_CO_3_ was used in order to compensate for the loss of Li) were mixed by hand grinding for 1 h. The resulting mixture was heated at 900 °C for 15 h and then 950 °C for 15 h. The heating and cooling rates were maintained at 2 °C min^−1^ ^46^. Figure S23 shows the morphology and the three-dimensional reconstruction of the as-synthesized Li_2_Ru_0.5_Mn_0.5_O_3_ material.

### Electrochemical measurement

The composite cathode was prepared by slurring the active material (Li_2_Ru_0.5_Mn_0.5_O_3_), carbon black, and polyvinylidene fluoride (PVDF) with a weight ratio of 8:1:1 in N-methylpyrrolidone solvent. The slurry mixture was then coated onto an aluminum current collector. High-purity lithium foil was used as the anode.

### Synchrotron X-ray pair distribution function (PDF) measurement

Total scattering pair distribution function (PDF) experiments were carried out at beamline 28-ID-2 in NSLS-II of BNL using an X-ray energy of 66.7 keV (*λ* = 0.186 Å) and an amorphous silicon area detector (Perkin-Elmer) to obtain data to large momentum transfer values. Data were integrated using the program Fit2D^47^. PDFgetX3^48^ was used to correct the data for background contributions, Compton scattering and detector effects, and to Fourier transform (*Q*_max_ = 23.5 Å) the data to generate the pair distribution function, G(*r*).

### TEM imaging and spectroscopy

The atomic-resolution scanning transmission electron microscopy (STEM) imaging of the LRMO cathode material was performed on Hitachi HD2700 (200 keV), JEOL Grand ARM (300 KeV), and FEI Talos F200X (200 keV) in a high-angle annular dark-field mode. The electron energy loss spectra were collected by Gatan Enfina and Enfinium spectrometers.

### X-ray photoemission spectroscopy

Our X-ray photoelectron spectroscopy (XPS) experiments were carried out in an ultrahigh vacuum (UHV) system with base pressures <2 × 10^−9^ Torr equipped a hemispherical electron energy analyzer (SPECS, PHOIBOS 100) and twin anode X-ray source (SPECS, XR50). Mg K_α_ (1253.6 eV) radiation was used at 10 kV and 30 mA. The angle between the analyzer and X-ray source is 45° and photoelectrons were collected along the sample surface normal.

### Electron tomography

The annular dark-field STEM (ADF-STEM) tomography tilt series were acquired on an uncorrected scanning/transmission electron microscope with an X-FEG field emission source (FEI Talos F200X). Projection images were acquired from −70 degrees to +70 degrees with two-degree tilt intervals. The chemical sensitive tilt series were acquired on the same instrument using the spectroscopic signals collected by a Bruker Super-X energy dispersive X-ray spectroscopy (EDX) detector. Projection STEM-EDX maps of ruthenium and manganese were acquired from −70 degrees to +70 degrees with ten-degree tilt intervals. The tomograms were reconstructed using a custom-written Matlab script implementing the multiplicative simultaneous iterative reconstruction technique. The three-dimensional reconstructions are visualized by Avizo.

### Full convex-hull phase diagram calculation

The phase diagram of Ru-Mn-O was calculated using the Phase Diagram app of the Materials Project^49^. In this project, the total energy of compounds is calculated using the density-functional theory as implemented in the Vienna Ab Initio Simulation Package (VASP) software. For the exchange-correlational functional, a mixture of Generalized Gradient Approximation (GGA) and GGA + U are and U values for many transition metals of interest have been calibrated using the approach outlined in Wang et al.‘s work^50^.

### Ab initio molecular dynamics simulation

The Ab Initio molecular dynamics (AIMD) simulation was carried out by Vienna ab Initio Simulation Package (VASP). The Perdew, Burke and Ernzerhof functional3 functional is used to describe the electron–electron exchange-correlation interaction with a plane-wave basis set. The 2 × 2 × 2 supercell of RuMnO_2_ with rock-salt structure is modeled as the starting structure. The canonical ensemble is used with Nosé-thermostat4 and the temperature in AIMD is 300 K. The time step is 0.5 fs with the total 1.3 ps. Spin polarization was not included in AIMD and formation energy calculations.

### Formation energy calculation

Self-consistent field, first-principles plane-wave calculations within density-functional theory (DFT) were performed for total energy and optimized structure calculations, as implemented in Quantum Espresso simulation package^51^. The calculations made use of the spin-dependent generalized gradient approximation of Perdew, Burke, and Ernzerhof (PBE)^52^, including Hubbard-U corrections following the formalism developed by Dudarev et al^53^. The wave function was expanded in a plane-wave basis set with an energy cutoff of 650 eV, and the unit cell and atomic positions of all structures in the delithiation were optimized until the atomic forces were less than 0.025 eV/angstrom. The electron states were sampled using a k-point mesh of 4 × 4 × 2 centered at the origin. For details, please see Supplementary Materials.

## Supplementary information

Supplementary Information

Description of Additional Supplementary Files

Supplementary Movie 1

Supplementary Movie 2

Supplementary Movie 3

Supplementary Movie 4

Supplementary Movie 5

Supplementary Movie 6

Supplementary Movie 7

## Data Availability

The custom-written code used in this study is available upon request.

## Data availability

The data supporting the findings of this study are available within the article and its Supplementary Information files, or from the corresponding authors on reasonable request.
