# Supplementary material for: Anomalous metal segregation in lithium-rich material provides design rules for stable cathode in lithium-ion battery
Source: Nat Commun. 2019 Apr 9;10:1650. doi: 10.1038/s41467-019-09248-0 (PMC6456622; doi:10.1038/s41467-019-09248-0)
Supplement: Supplementary file 1 — Supplementary Information [file 41467_2019_9248_MOESM1_ESM.pdf]

Supplementary Information for

Anomalous metal segregation observed in lithium-rich layered oxide provides new concepts in theoretical design for stable cathode in lithium-ion battery

Lin *et al*.

Supplementary Figures

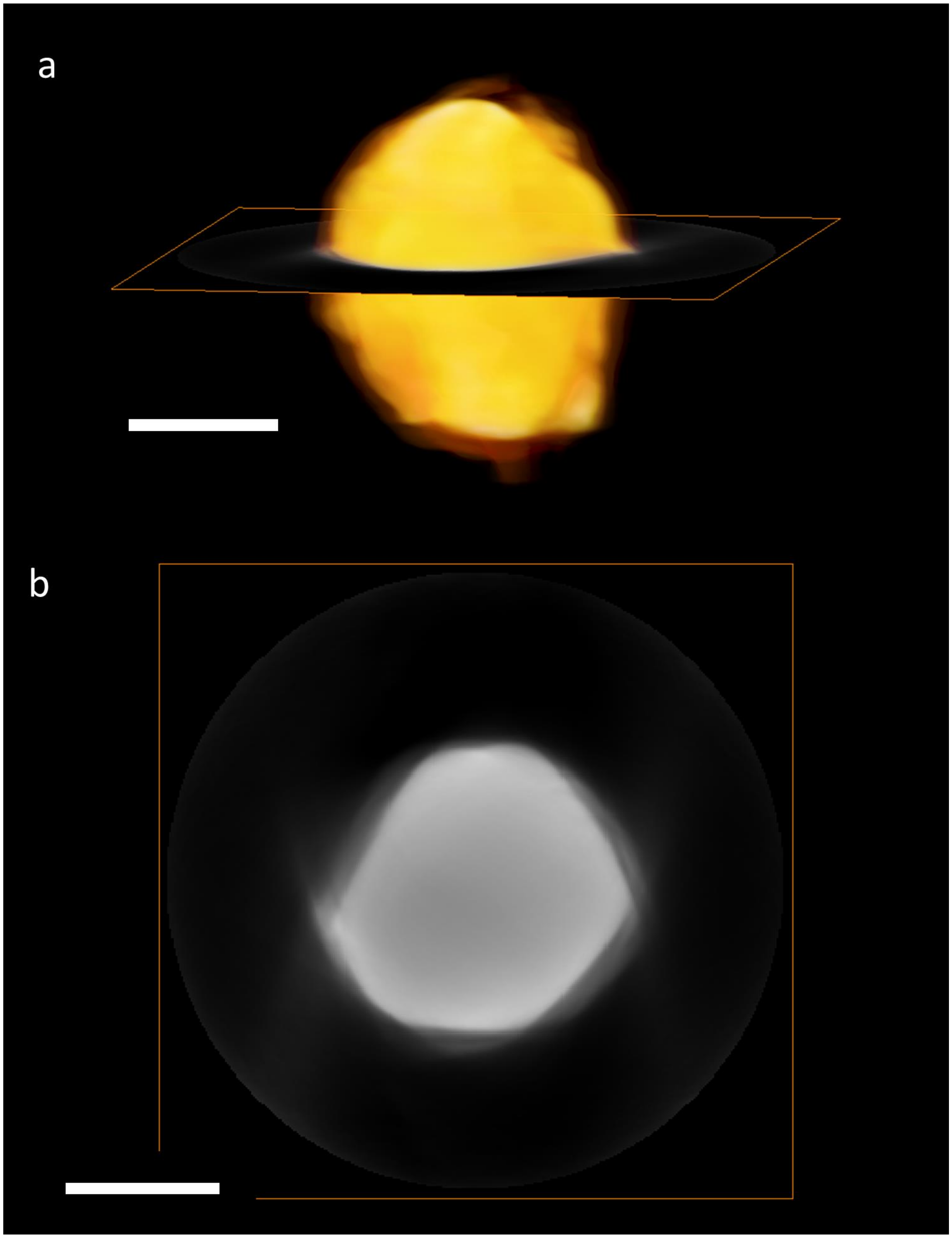

Supplementary Figure 1. Electron tomography of pristine LRMO particle. (a) The volume rendering of a primary particle. (b) A cross-sectional image of the 3D reconstruction showing

that the interior of the particle is solid and has no pores. The full cross-sectional movie of the reconstruction is shown in Supplementary Movie 2.

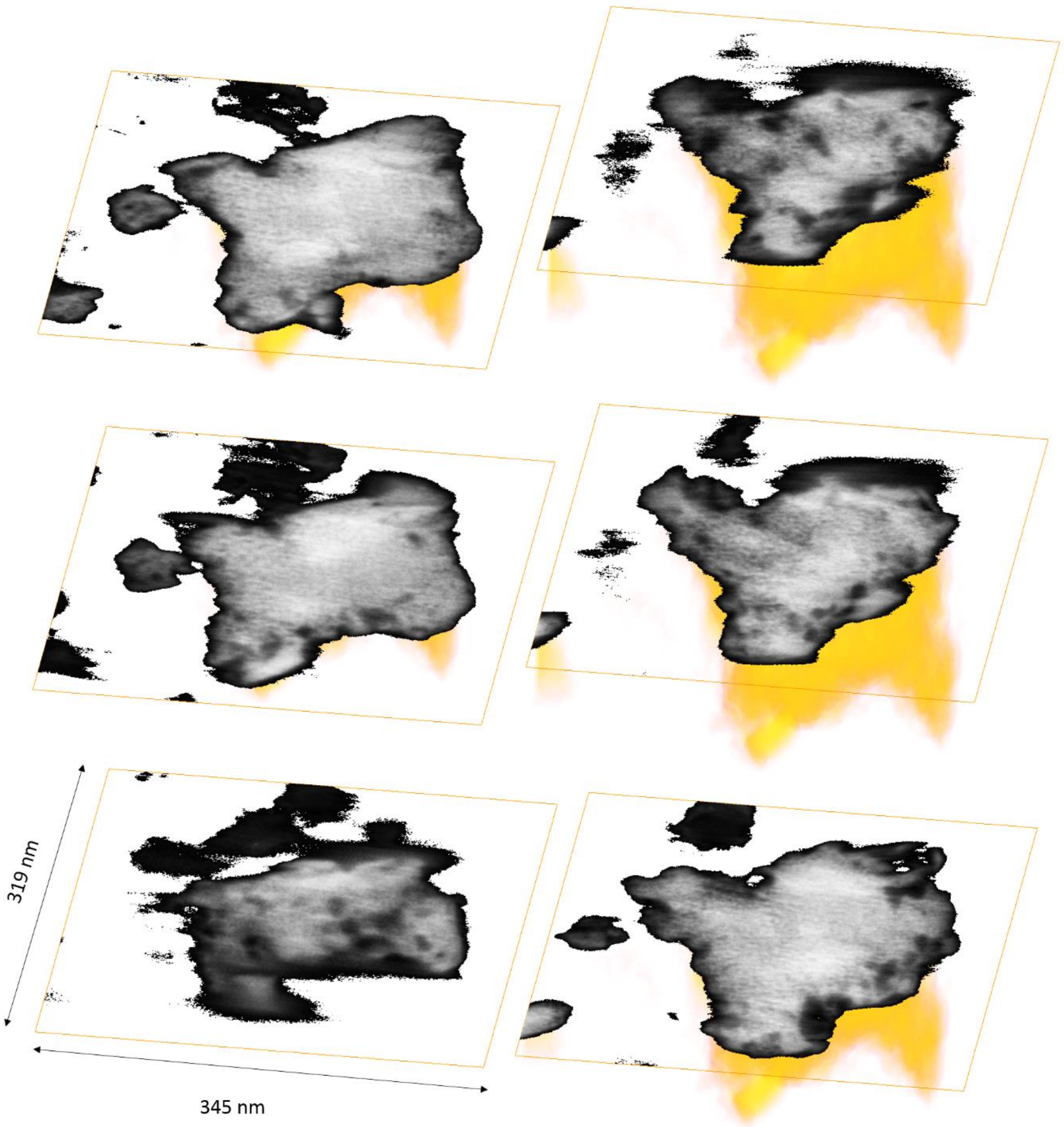

Supplementary Figure 2. Progressive cross-section view of the internal porous structure of LRMO after 97 cycles.

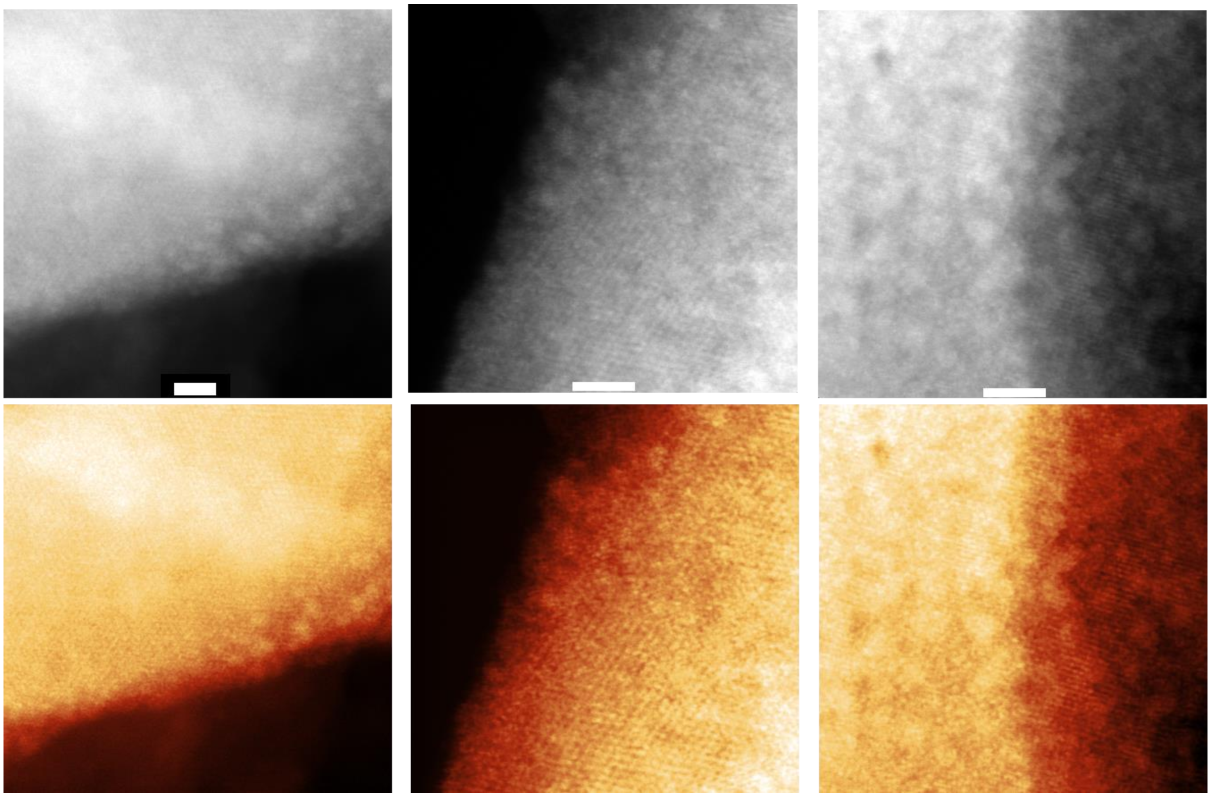

Supplementary Figure 3. Z-contrast STEM Image of the LRMO surfaces after one charge/discharge cycle (OCV-4.6V-2V) showing formation of particles. These images were recorded in a FEI Talos operated at 200 keV and equipped with an extra-bright field emitter (X-FEG). (Scale bar: 3 nm)

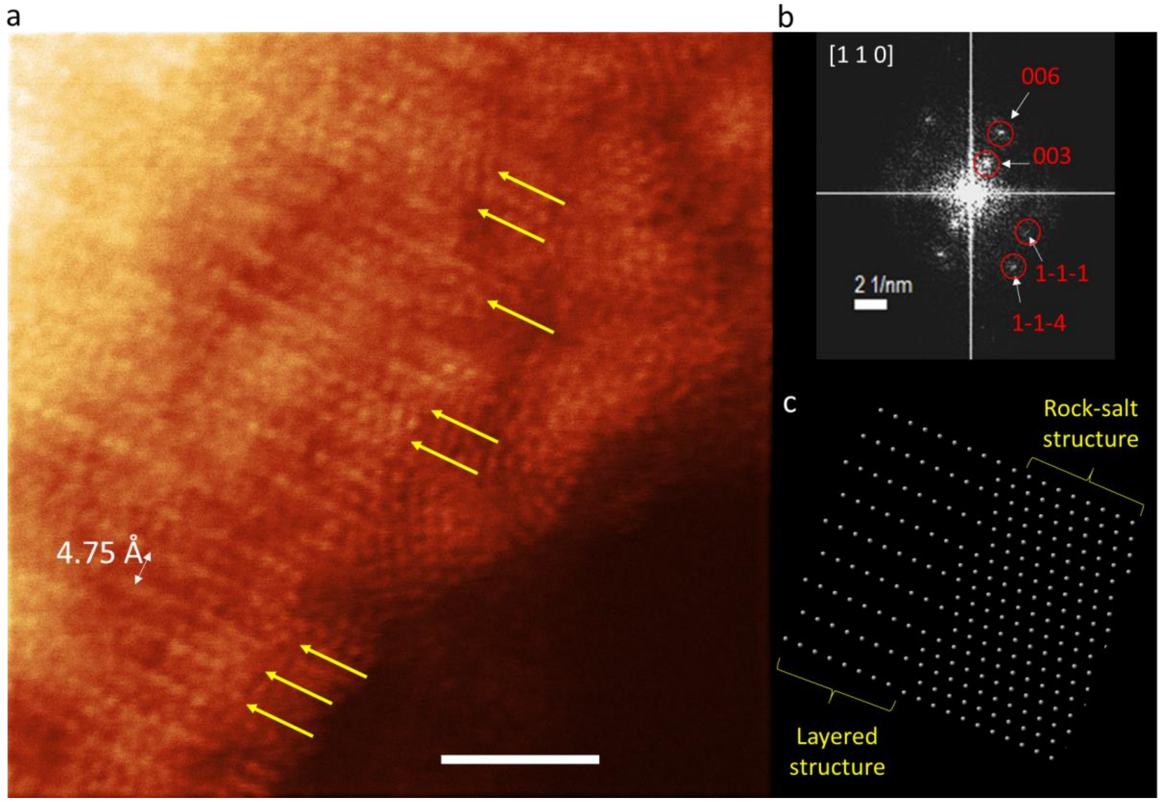

Supplementary Figure 4. Z-contrast STEM imaging. (a) Atomic-resolution Z-contrast STEM imaging of LRMO surface after 15 cycles. The layer to layer distance is measured to be 4.75±0.05 Å. The arros points to the locations where the lithium channels are filled with higher atomic number transition metals. These are indicative of reconstructed rock-salt formation. (b) The zone axis is indexed to be the [1 1 0] direction in the simplified R-3m structure. (c) The atomic model projected along the [1 1 0] zone axis showing what the projected image would look like when a rock-salt reconstruction layer is formed. (Scale bar: 2 nm)

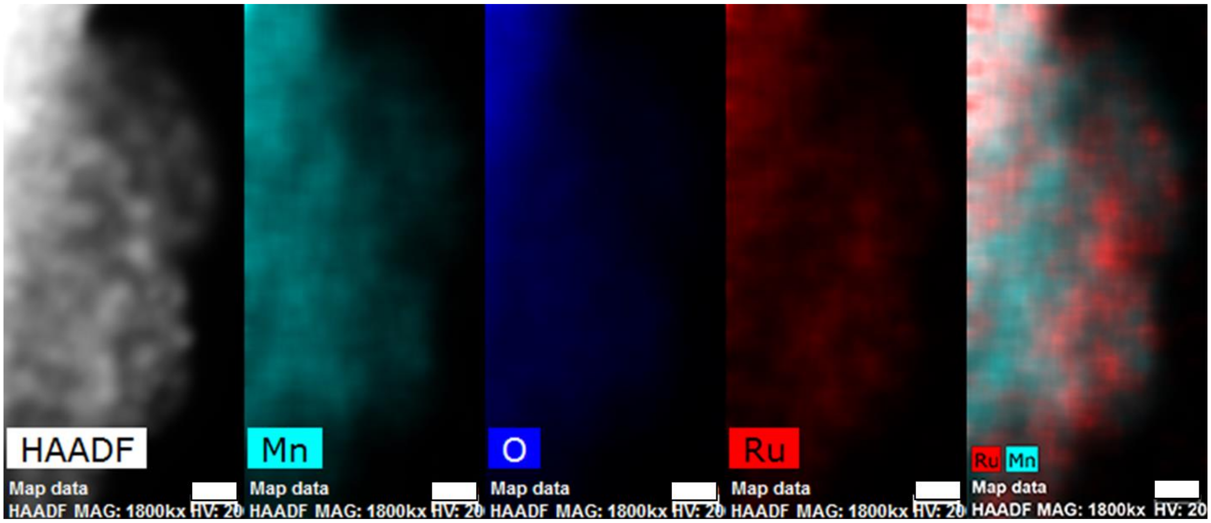

Supplementary Figure 5. STEM-EDX mapping showing the high-intensity clusters are rich in Ru. These results were recorded in a FEI Talos operated at 200 keV and equipped with an extra-bright field emitter (X-FEG) and a Bruker four-quadrant energy dispersive X-ray spectrometer. (Scale bar: 3 nm)

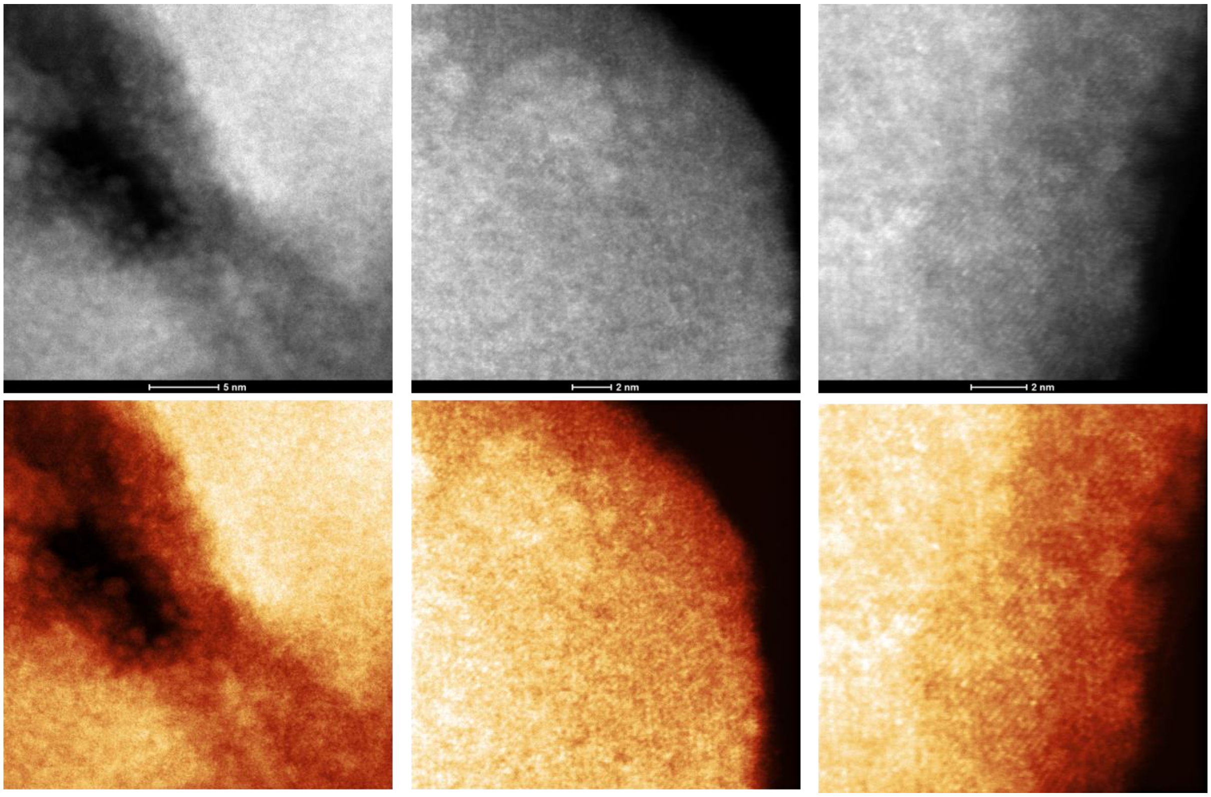

Supplementary Figure 6. Z-contrast STEM Image of the LRMO surfaces after one charge/discharge cycle (OCV-4.6V-3.5V) showing that the formation of ruthenium clusters is not due to discharging down to a lower cut-off voltage of 2 V.

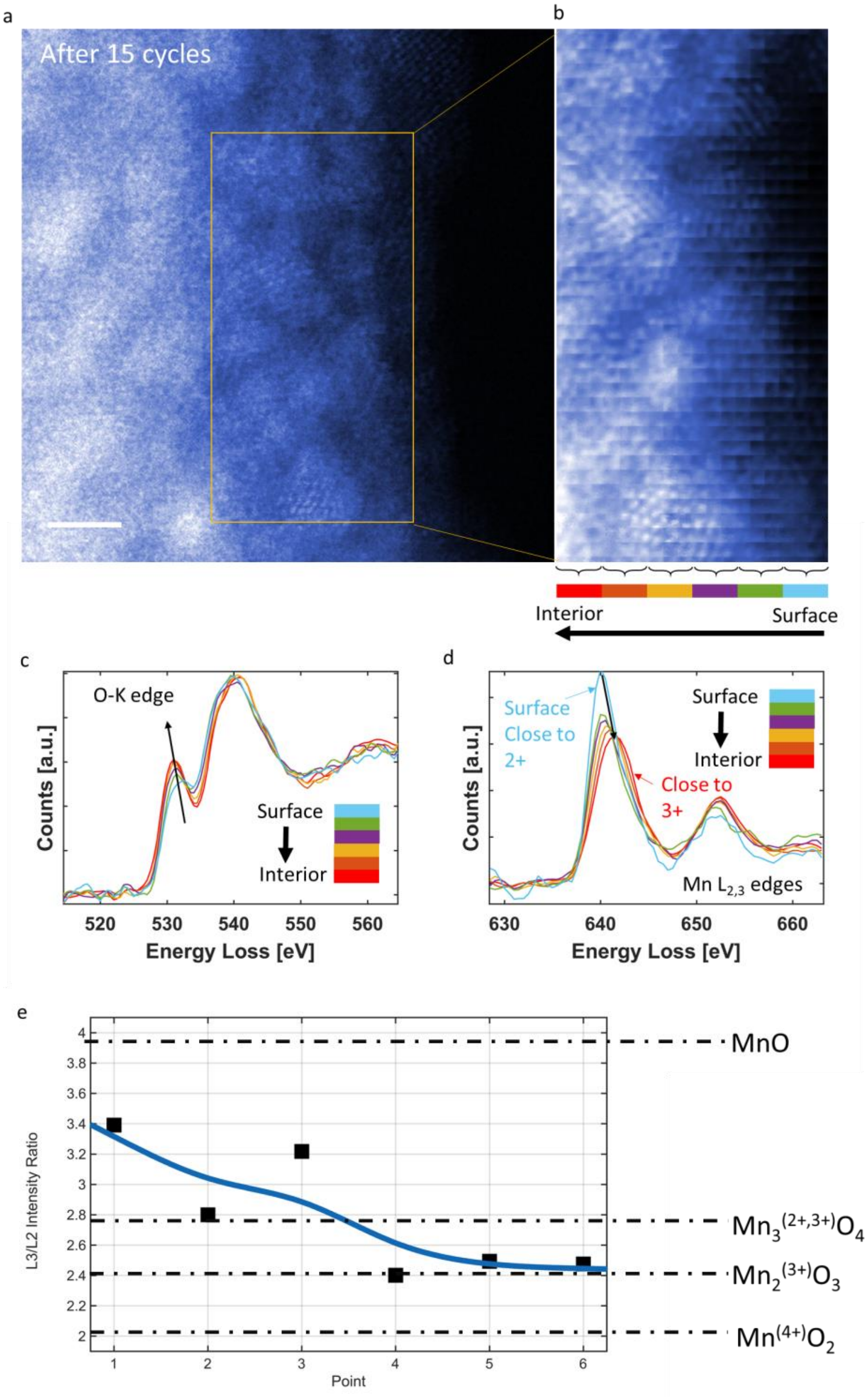

Supplementary Figure 7. STEM-EELS mapping of the surface and sub-surface area of a LRMO particle after 15 cycles. (a) Z-contrast STEM image of the surface of a primary particle. (b) Z-contrast STEM image simultaneously acquired with EELS mapping. To spread dose and reduce damage, at each EELS acquisition pixel, the electron beam sub-scanned within the pixel

area and a 16×16-pixel sub-scanned image was taken while EELS signal was integrated. The final image was formed by montaging the sub-scanned images together. (c, d) EELS spectra of (c) O-K edge and (d) Mn L2,3 edges from the surface to the interior. (c) O K-edge spectra show the pre-edge peaks intensity increases as the probed area moves away from the surface indicating the metal on the surface has a lower valence state. Here the pre-peak intensity is associated with the transitions from O 1*s* to O 2*p*-metal 3*d* hybridized states and tracks the number of d-holes of the nearest-neighbor transition metals. (d) Mn $L_{2,3}$ spectra showing that the L3 white line of Mn moves lower energy as the probed area moves to the surface. At the same time, the L3 intensity increase with respect to the decreasing L2 intensity—L3/L2 ratio decreases. All these points to Mn takes a lower valence on the surface[1]. (e) L3/L2 intensity ratio analysis of Mn L2,3 edges. We can approximately conclude the surface spectrum represent a Mn valence close to Mn^{2+} and the interior spectrum approximately represent Mn^{3+}. The reference ratios are adapted from the following reference[2]. (Scale bar: 2 nm)

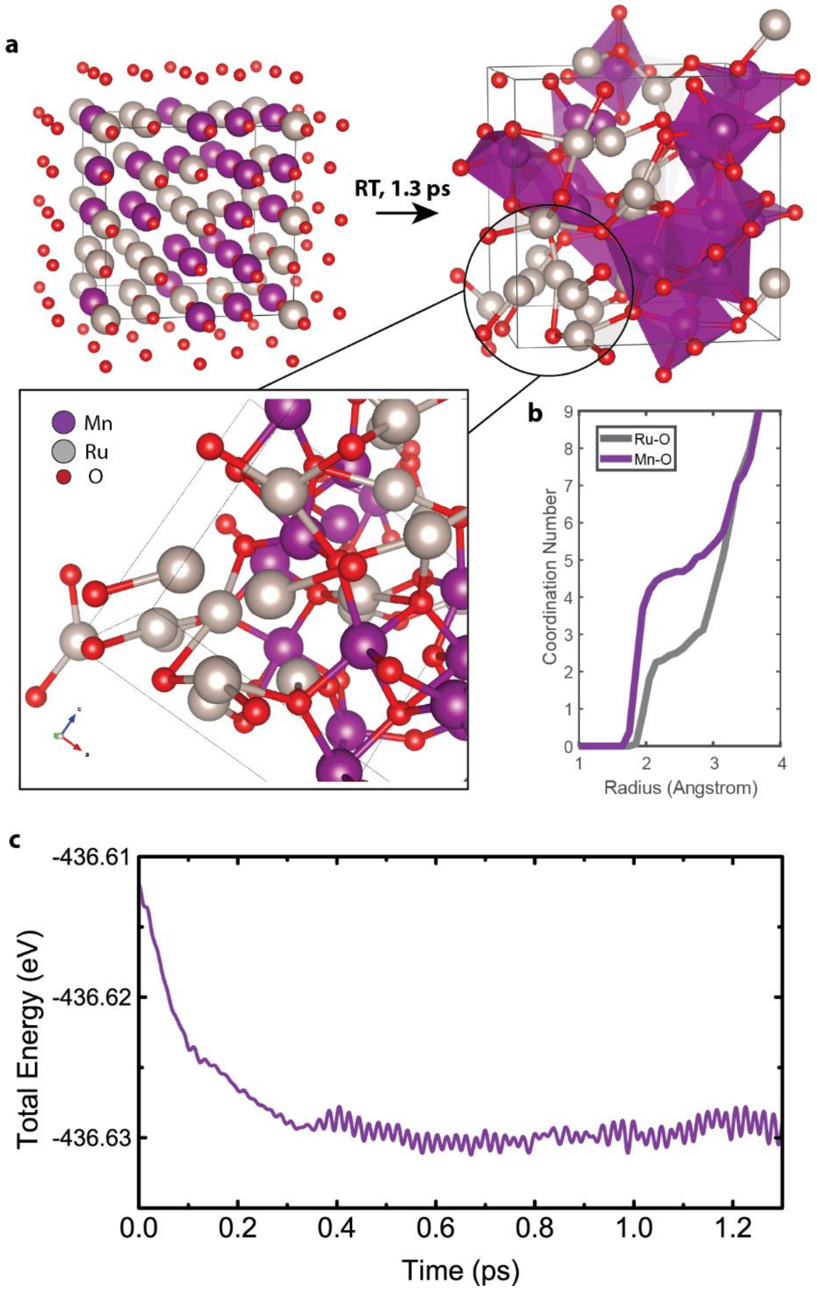

Supplementary Figure 8. Ab initio MD simulation. **a**, Ab inito molecular dynamic (MD) simulation of the room temperature instability of a $Ru_{0.5}Mn_{0.5}O$ reconstructed layer. The 1.3-ps structure shows that Ru tend to segregate and repels oxygen to the neighborhood of Mn. **b**, Ru-O and Mn-O coordination number as a function of radius. The Ru-O coordination number is approximately half that of Mn-O at a radius of 2.2 angstrom agrees with our qualitative observation in (a). **c**, Time evolution of the total energy of a 2×2×2 Metal-Oxygen (MO) rock-salt unit cell during the room-temperature MD simulation.

a

$$E_f = E_{\mathrm{Mn_xM_{1-x}O}} - x \cdot E_{\mathrm{MnO}} - (1-x) \cdot E_{\mathrm{MO}}$$

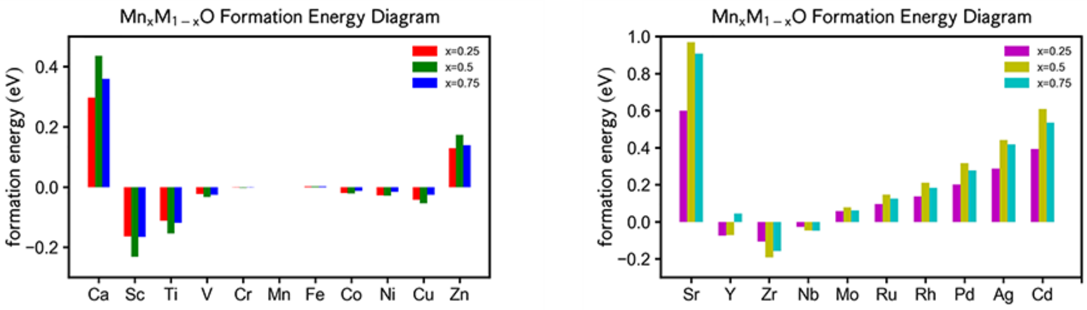

b

$$E_f = E_{\mathrm{Ni_xM_{1-x}O}} - x \cdot E_{\mathrm{NiO}} - (1-x) \cdot E_{\mathrm{MO}}$$

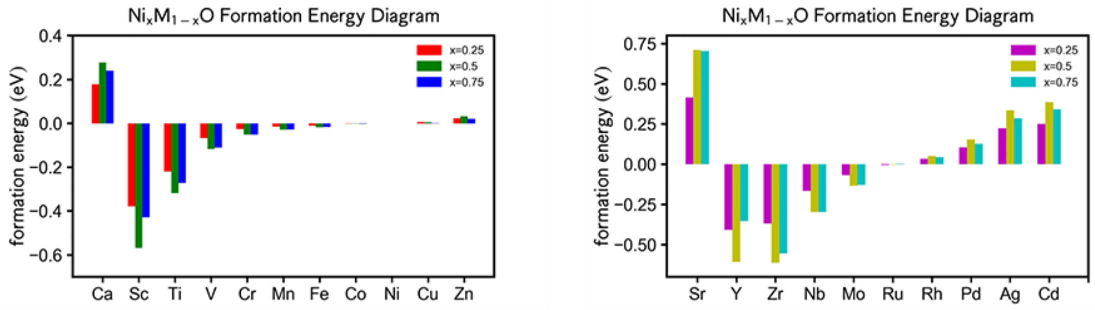

c

$$E_f = E_{\mathrm{Co_xM_{1-x}O}} - x \cdot E_{\mathrm{CoO}} - (1-x) \cdot E_{\mathrm{MO}}$$

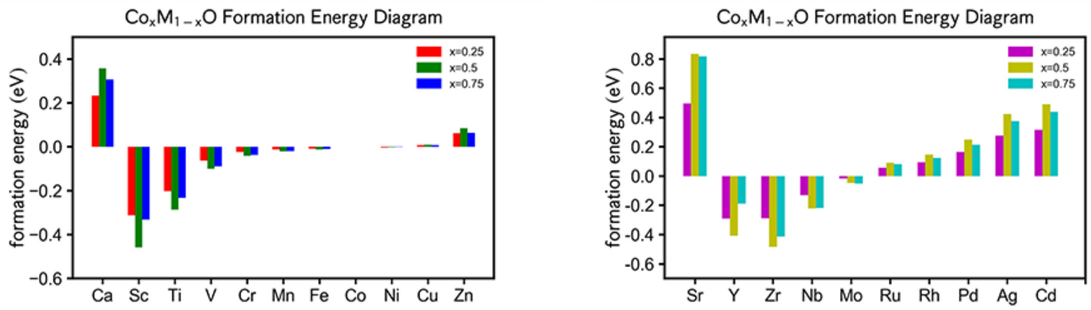

Supplementary Figure 9. The calculation of solubility of all 3*d*/4*d* transition metals in a $T_xM_{1-x}O$ (T = Mn, Ni, Co; M = 3*d*/4*d* species) rock-salt structure.

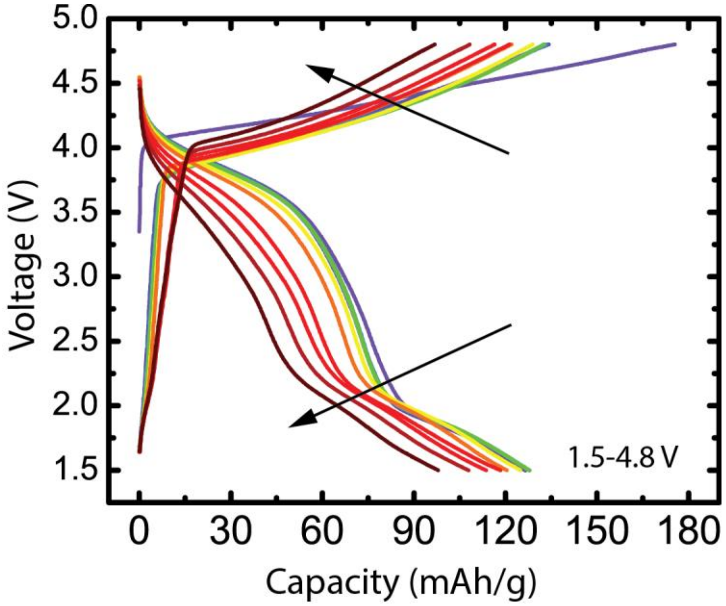

Supplementary Figure 10. Charge/discharge curves of LNTN/lithium half-cell with cut-off voltages of 1.5 V and 4.8 V over 50 charge/discharge cycles.

Pristine

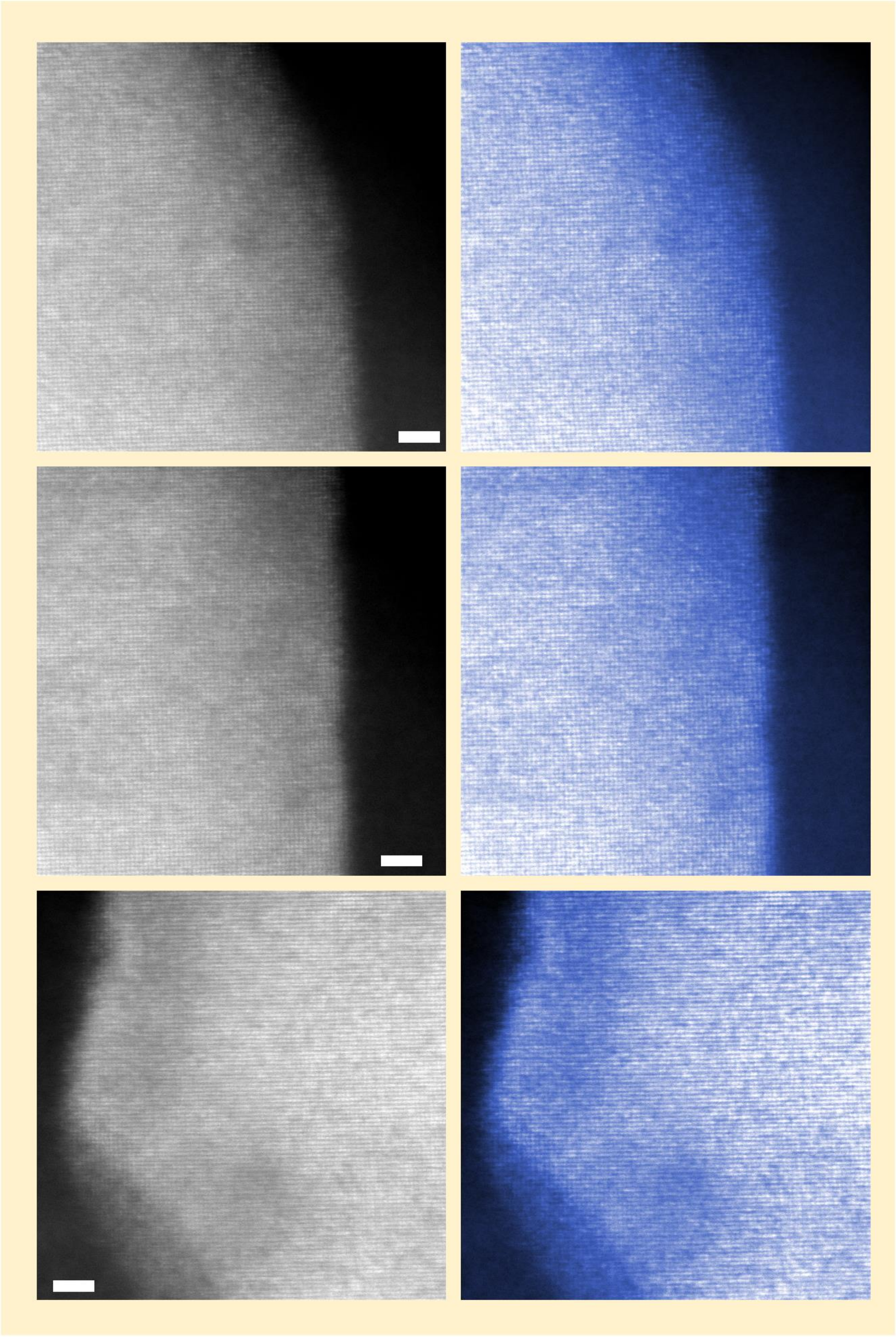

Supplementary Figure 11. Atomic-resolution Z-contrast STEM imaging of near-surface area of lithium-rich nickel-titanium-niobium oxide in its pristine state. (Scale bar: 2 nm)

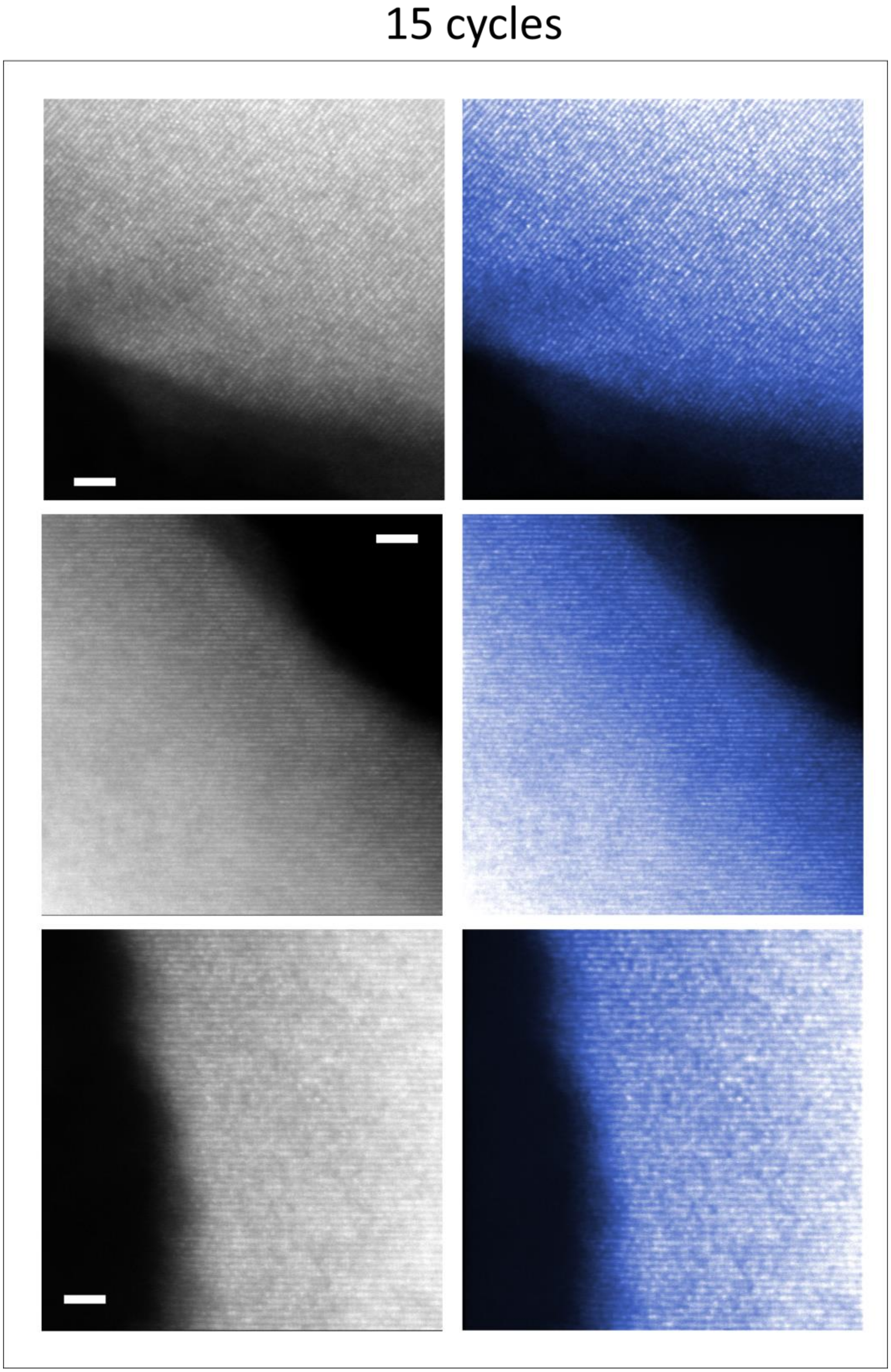

Supplementary Figure 12. Atomic-resolution Z-contrast STEM imaging of near-surface area of lithium-rich nickel-titanium-niobium oxide after 15 cycles. The variance of atomic column

intensity of the cycled sample is comparable to that of the pristine sample and no segregation of niobium or niobium oxide is observed. (Scale bar: 2 nm)

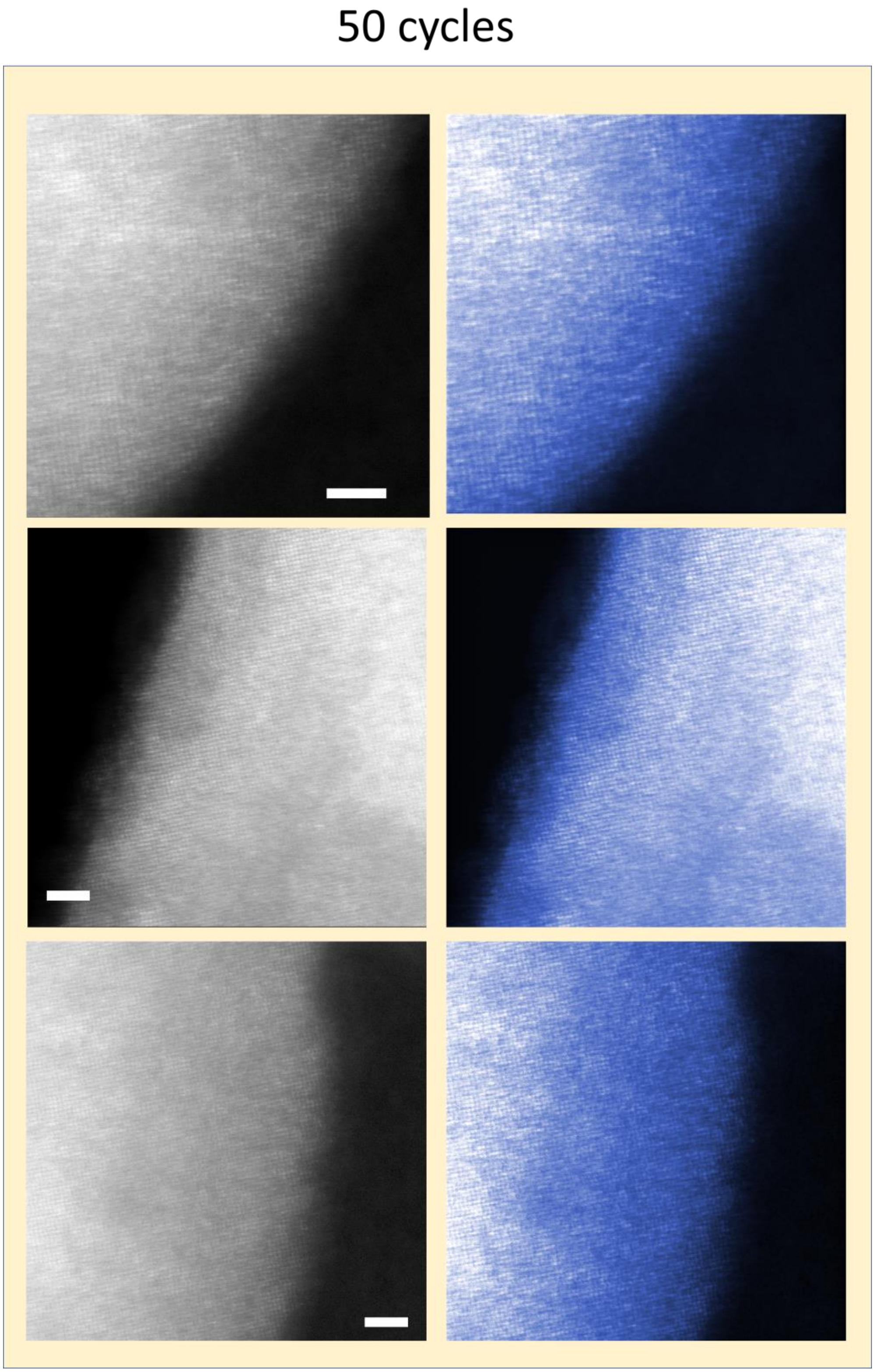

Supplementary Figure 13. Atomic-resolution Z-contrast STEM imaging of near-surface area of lithium-rich nickel-titanium-niobium oxide after 50 cycles. The variance of atomic column

intensity of the cycled sample is comparable to that of the pristine sample and no segregation of niobium or niobium oxide is observed. (Scale bar: 2 nm)

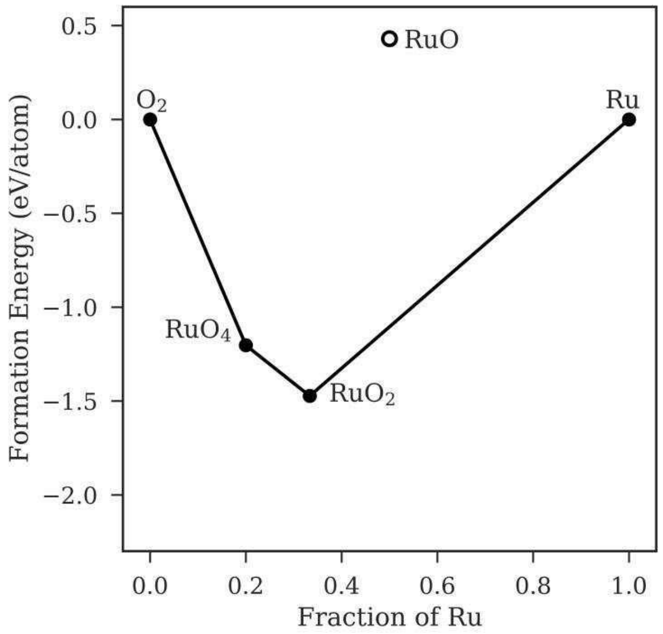

Supplementary Figure 14. The convex hull construction of Ru-O showing that rock-salt RuO is above the convex hull line indicating it is not a stable phase.

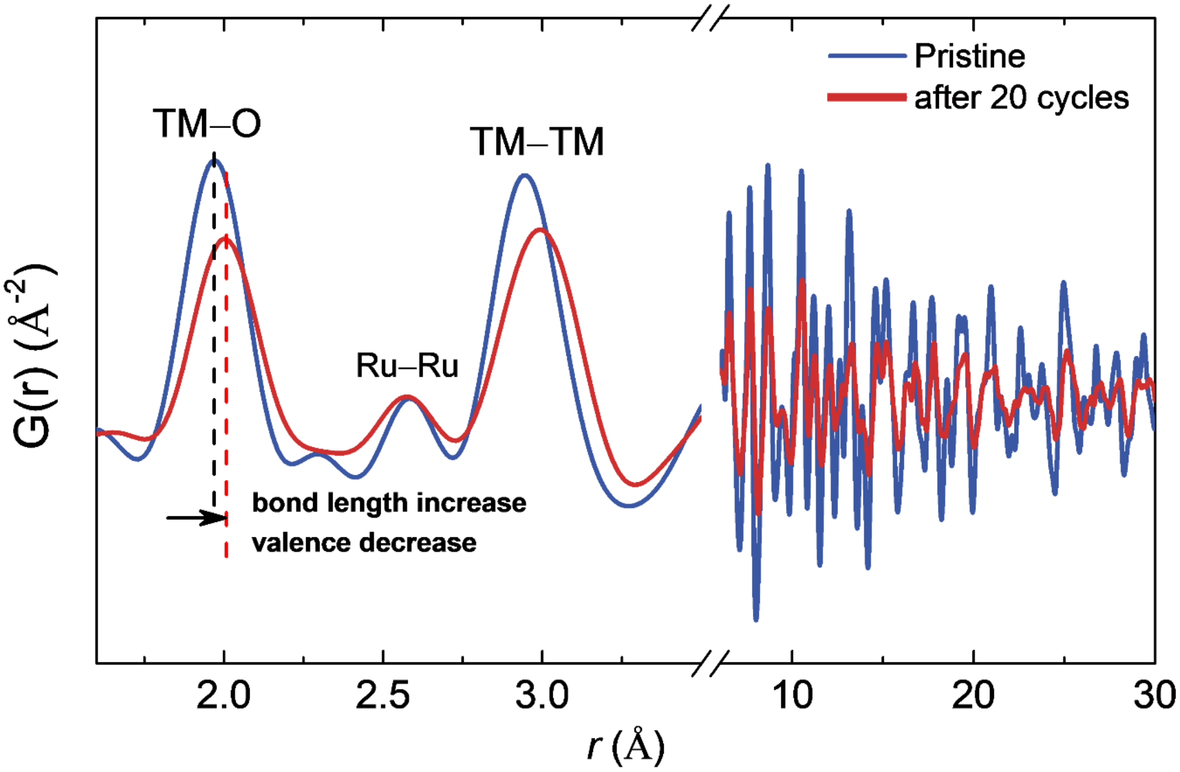

Supplementary Figure 15. The extended view of the PDF data shown in Figure 1 in the main text. We conclude that there is more disorder in the structure after cycling based on the comparison of PDF data for both cycled sample and pristine sample. It clearly shows that as electrochemical cycle number increases, PDF peaks which corresponding to atomic pairs, are significantly broadened. This can be either due to enhanced thermal motion or more static disorder that leads to larger distribution of atomic positions. Since the temperature is not changed, it is most likely that such PDF peak broadening is due to structural disorder.

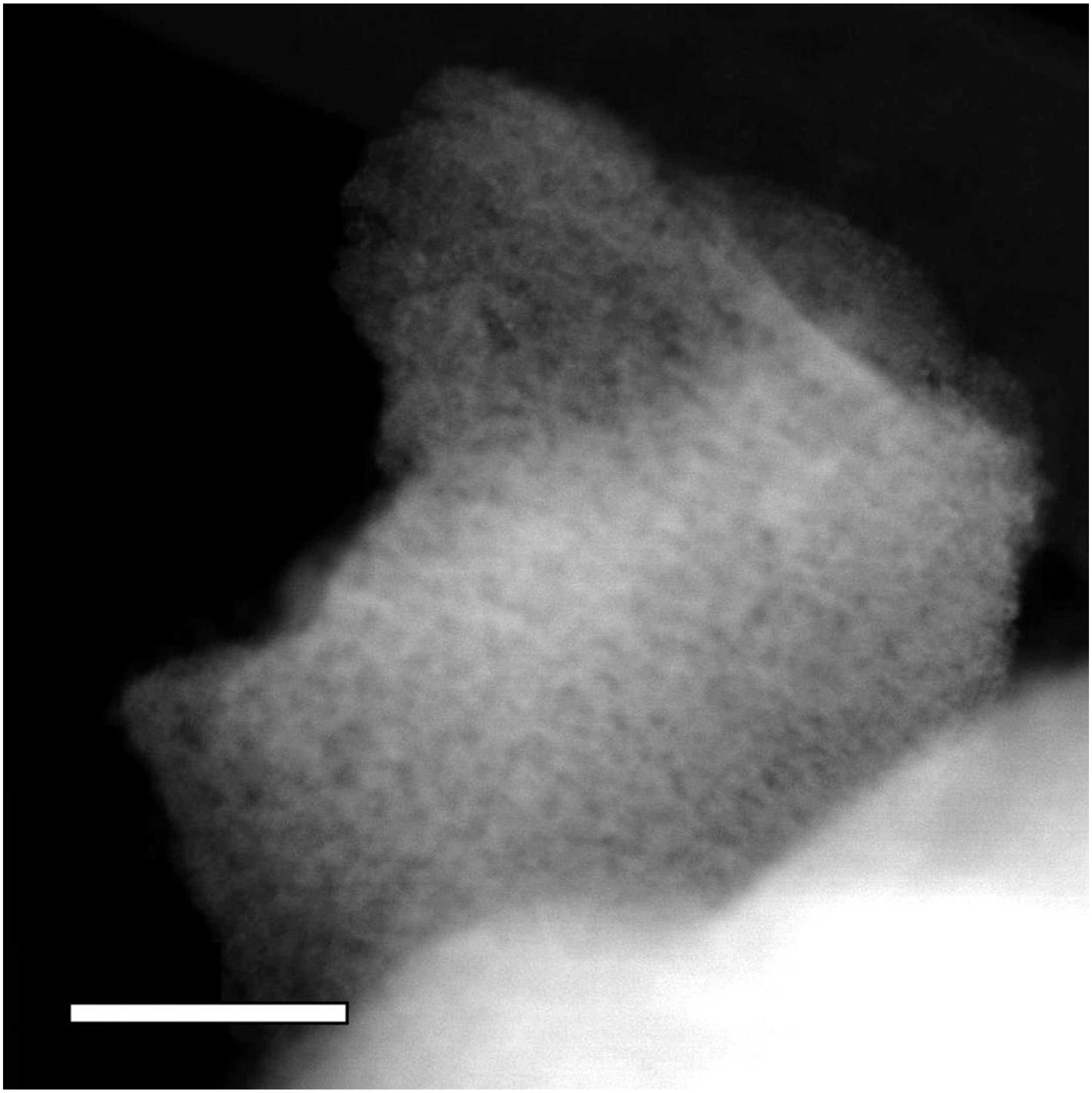

Supplementary Figure 16. Single projection image of the 97-cycle sample showing a porous structure has been developed. The image was taken with a gentle imaging condition (Electron dose<150 electron/Angstrom^2) (Scale bar: 50 nm)

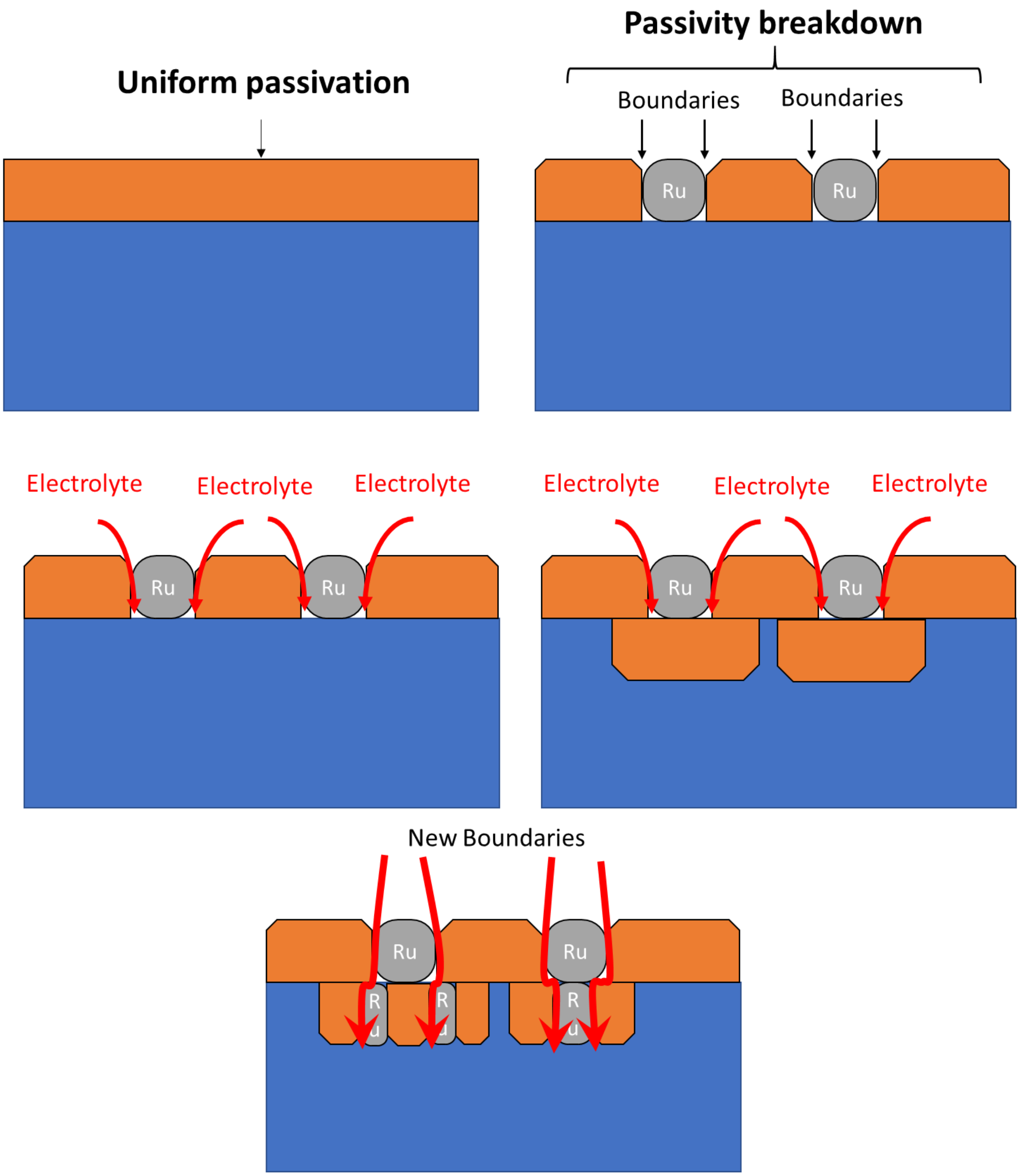

Supplementary Figure 17. The MnO passivation film is disrupted by the segregated Ru metal clusters that leaves **boundaries and grooves** that allow electrolyte to quickly permeate through and meet the undegraded interior. This process can happen in a recursive fashion like a domino chain reaction. This point is supported by Fig. 3b in the main text, the degraded surface is not uniform at all. To protect the surface, a uniform passivation film needs to form.

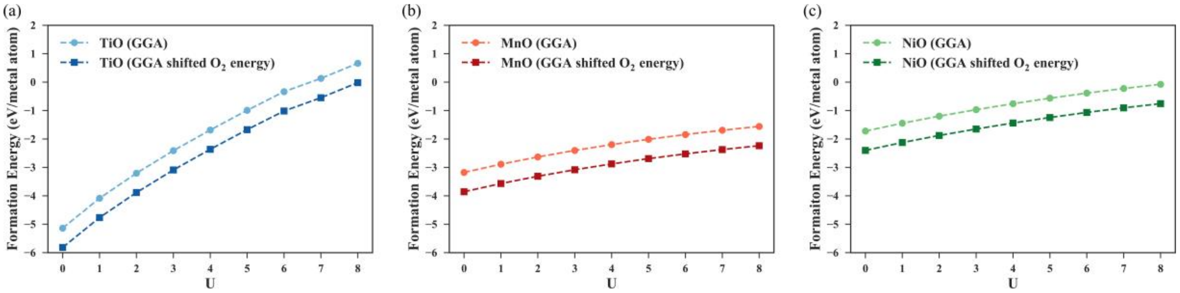

Supplementary Figure 18. Formation energy of (a)TiO, (b)MnO, (c)NiO varies with U parameter.

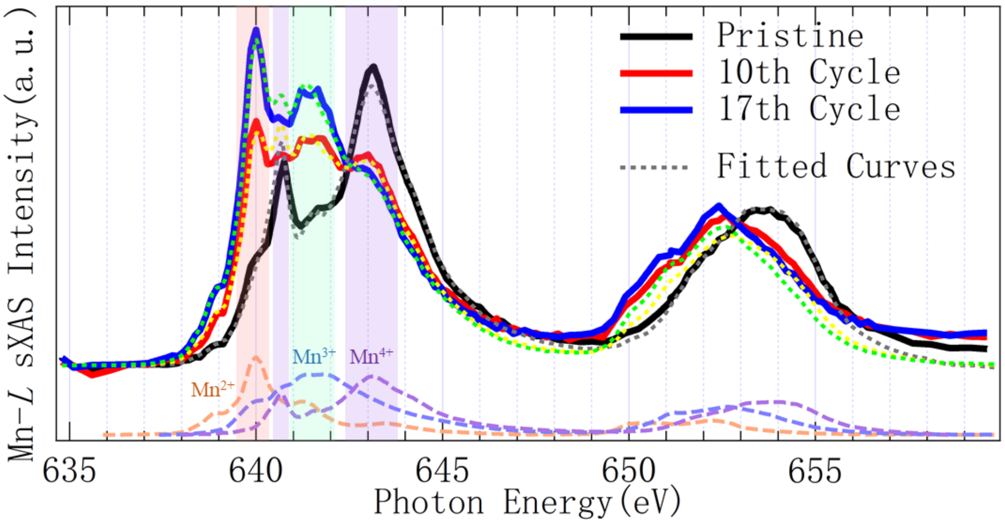

	Mn^{2+} (%)	σ*	Mn^{3+} (%)	σ	Mn^{4+} (%)	σ
Pristine	6.5	0.8	11.3	1.7	82.2	1.5
10th Cycle	22.0	0.6	44.1	1.3	33.9	1.2
17th Cycle	32.0	0.6	51.7	1.3	16.3	1.1

Supplementary Figure 19. XAS spectra of the pristine LRMO, and LRMO after 10 and 17 cycles measured using the total electron yield (TEY) mode. One can qualitatively see that the Mn^{2+} contribution to the L_3 spectrum increases on the surface as the cycle number increases. By fitting the Mn L_3 edge with a linear combination of $Mn^{2+,3+,4+}$ reference spectra, Mn^{2+}

component is increased from 6.5% to 22% after 10 cycles and 32% after 17 cycles, and Mn^{3+} component is increased from 11.3% to 44.1% after 10 cycles and 51.7% after 17 cycles.

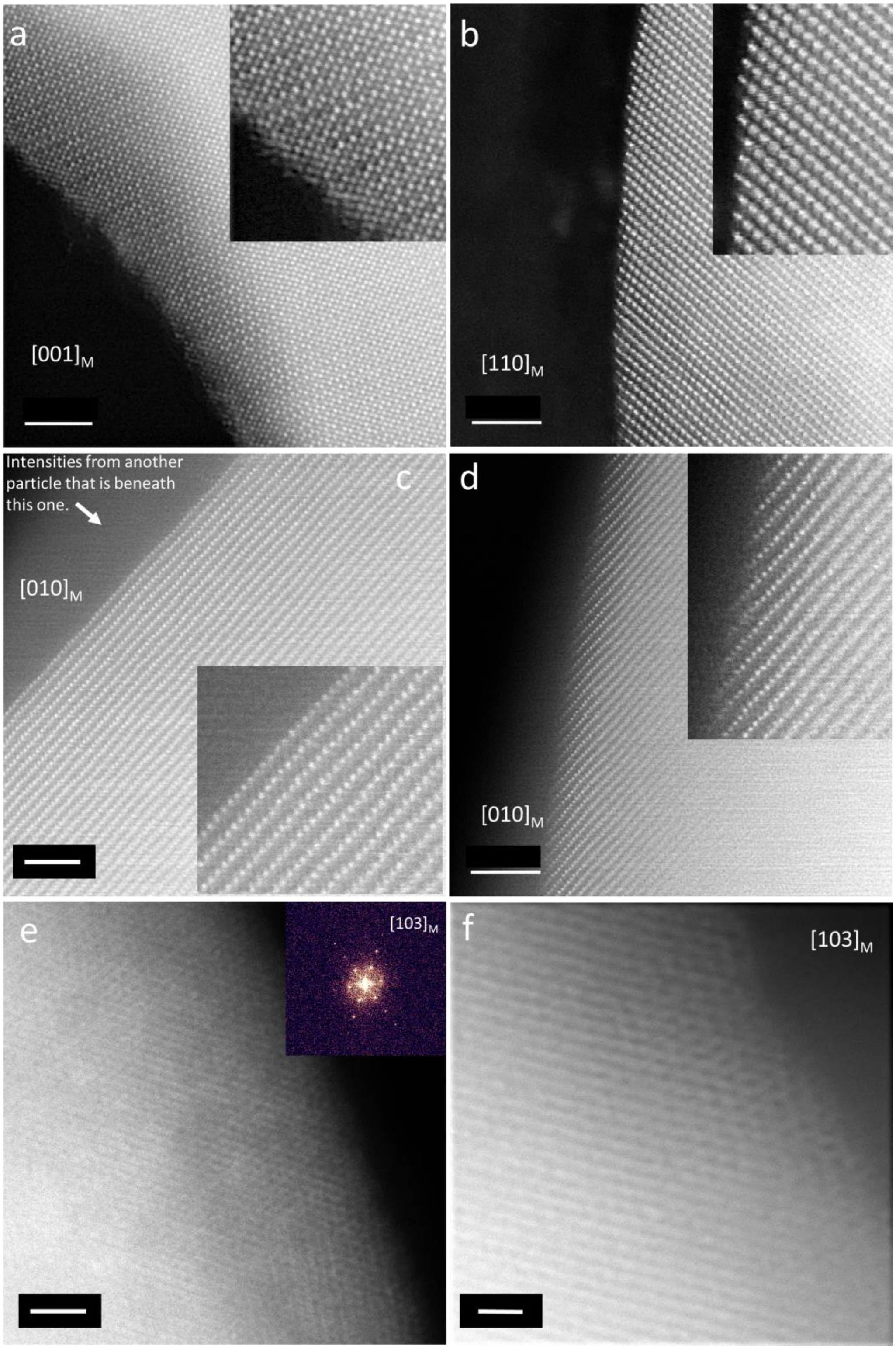

Supplementary Figure 20. The pristine LRMO material imaged down (a) $[001]_M$, (b) $[110]_M$, (c and d) $[010]_M$, and (e and f) $[103]_M$ zone axes. (a-d) Images were acquired in an aberration corrected 200 keV dedicated STEM. (e and f) Images were acquired by an uncorrected 200 keV S/TEM. The resolution of this instrument is not sufficient to resolve the atomic columns down the $[103]_M$ zone axis of LRMO, but the sub-lattice structure is resolved that shows the same lattice pattern extends all the way to the surface. (Scale bar: a. 3 nm, b. 4 nm, c. 3 nm, d. 4 nm, e. 3 nm, f. 1 nm)

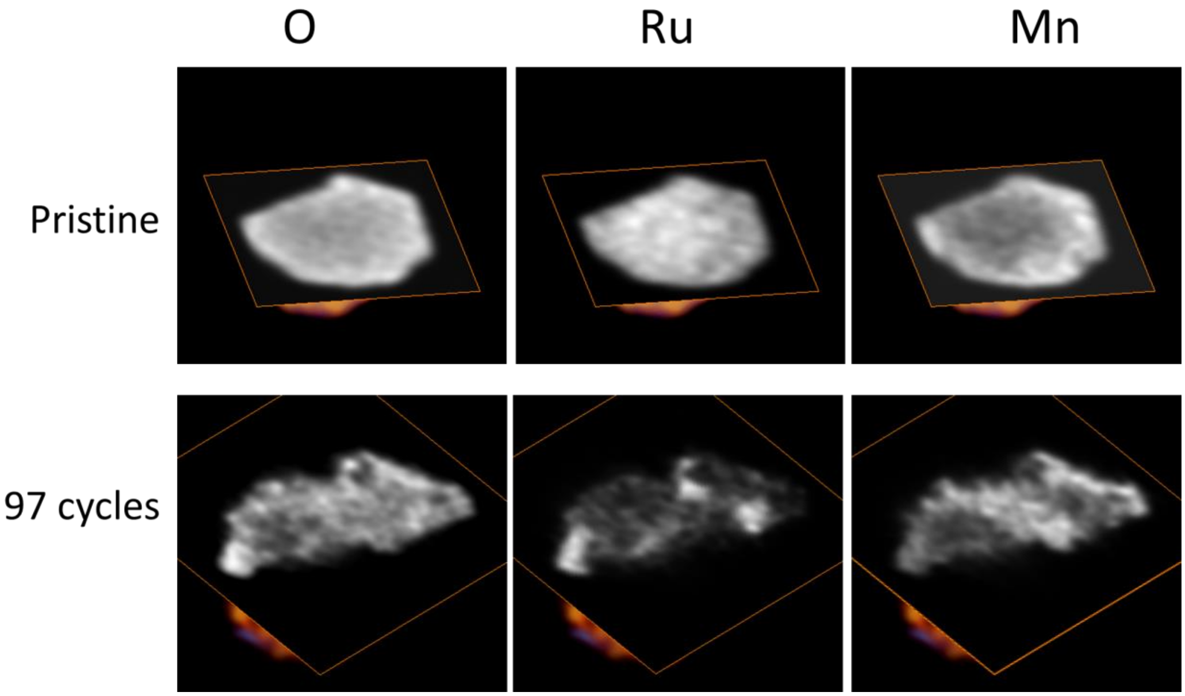

Supplementary Figure 21. Cross-sectional view of the chemical reconstruction of the LRMO material in (top) the pristine state, and (bottm) after extended cycling.

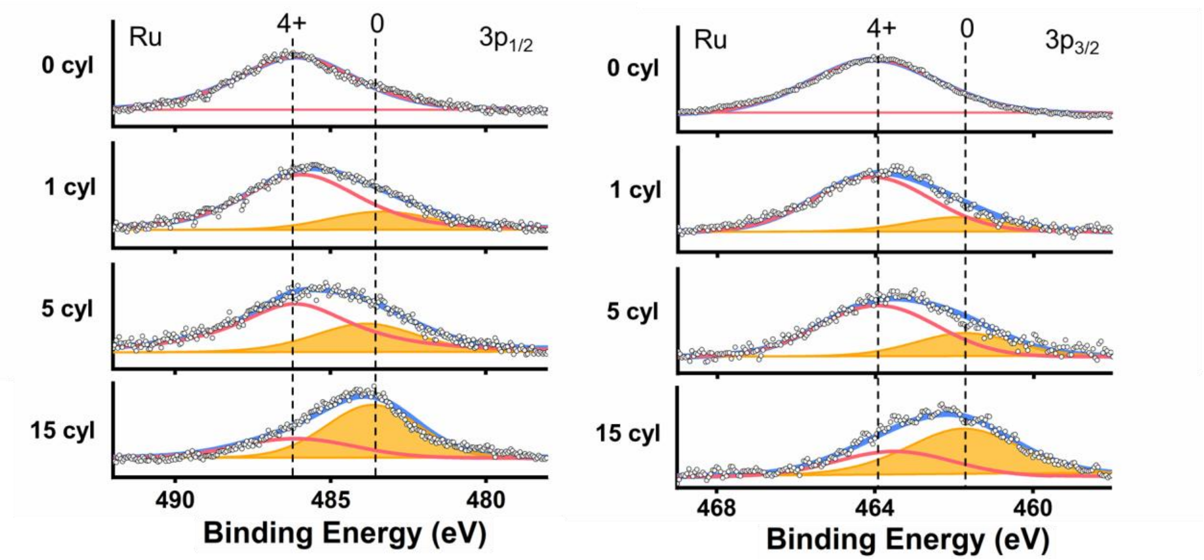

Supplementary Figure 22. Fitting of the Ru 2p1/2 and 2p3/2 XPS spectra.

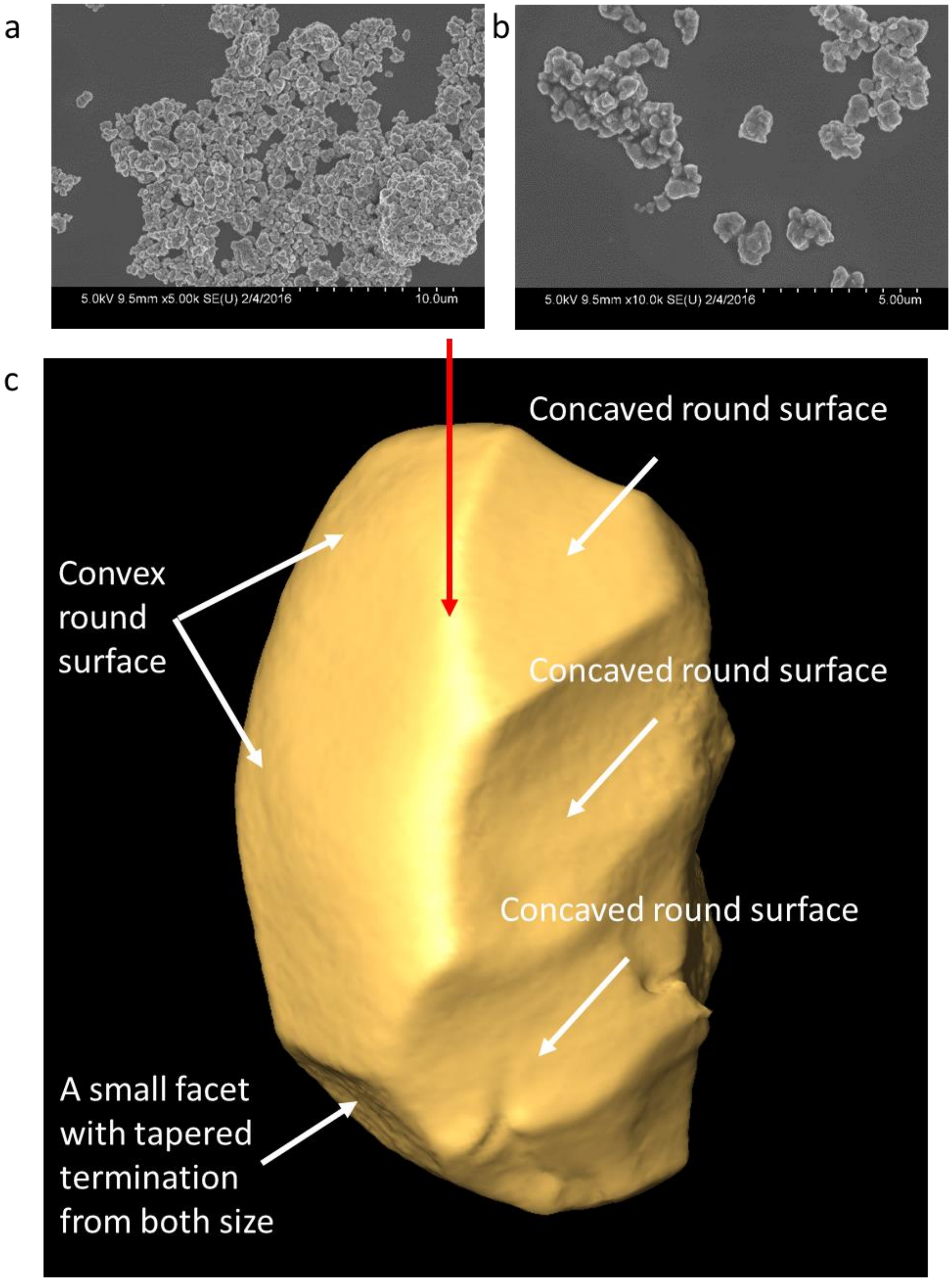

Supplementary Figure 23. SEM and 3D reconstruction images of the as-synthesized $Li_2Ru_{0.5}Mn_{0.5}O_3$. (a, b) SEM image of the as-synthesized $Li_2Ru_{0.5}Mn_{0.5}O_3$. (c) Electron tomography reconstruction shows that the primary particle has many curved surfaces.

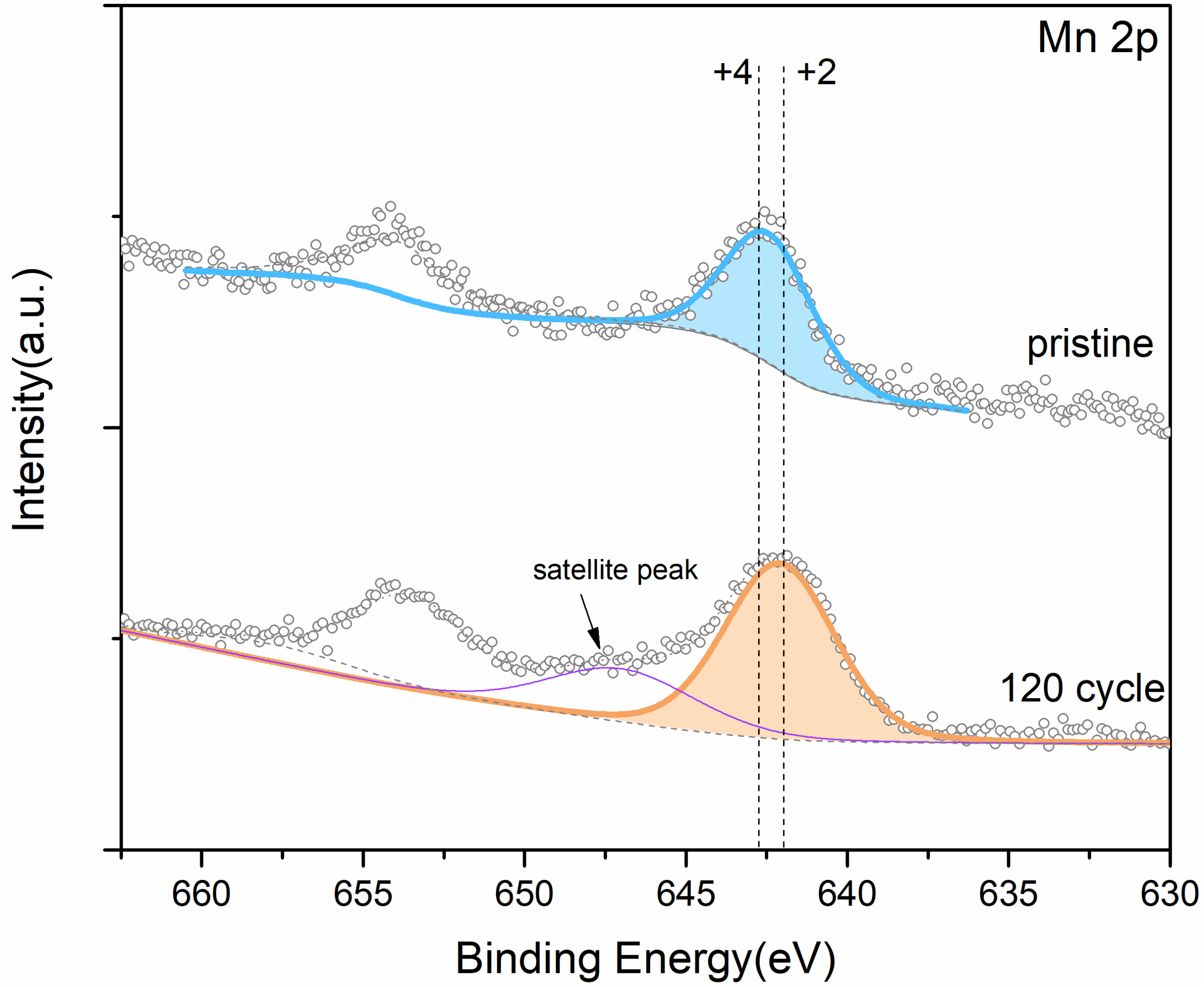

Supplementary Figure 24. SEM images of the as-synthesized $Li_2Ru_{0.5}Mn_{0.5}O_3$.

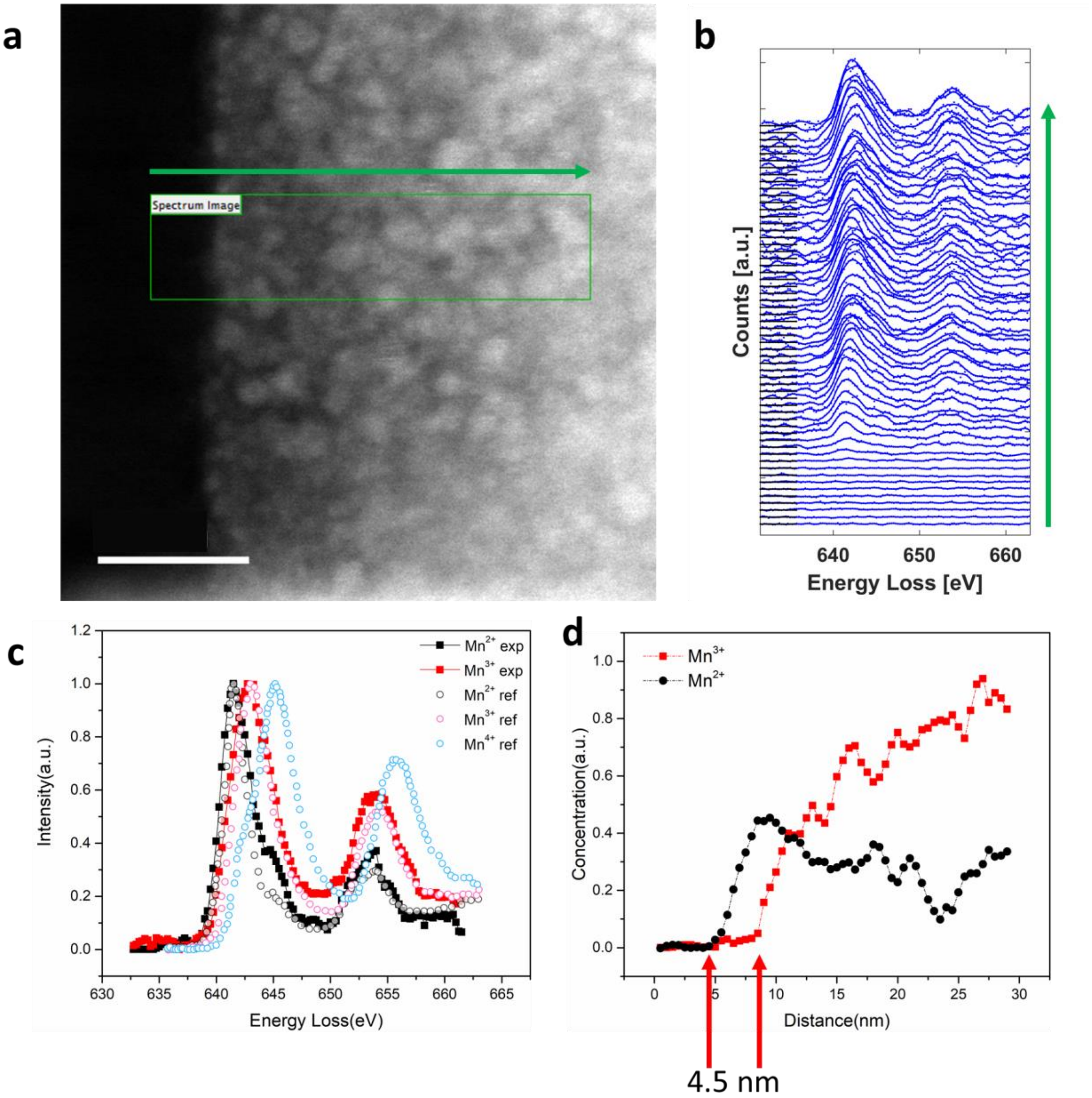

Supplementary Figure 25. STEM-EELS mapping of the 120-cycled LRMO surface. The top 4.5 nm surface region of the sample is nearly fully Mn^{2+}. (a) Mapping region. (b) EELS spectra. (c) Mn 2+, 3+, 4+ reference spectra (Tan et al, Ultramicroscopy 116 (2012) 24–33)[3] and the purified components of the experimental EELS spectra using Multivariate Curve Resolution. (d) Concentration profile of the Mn^{2+} and Mn^{3+} components from the surface to the interior of the particle. (Scale bar: 10 nm)

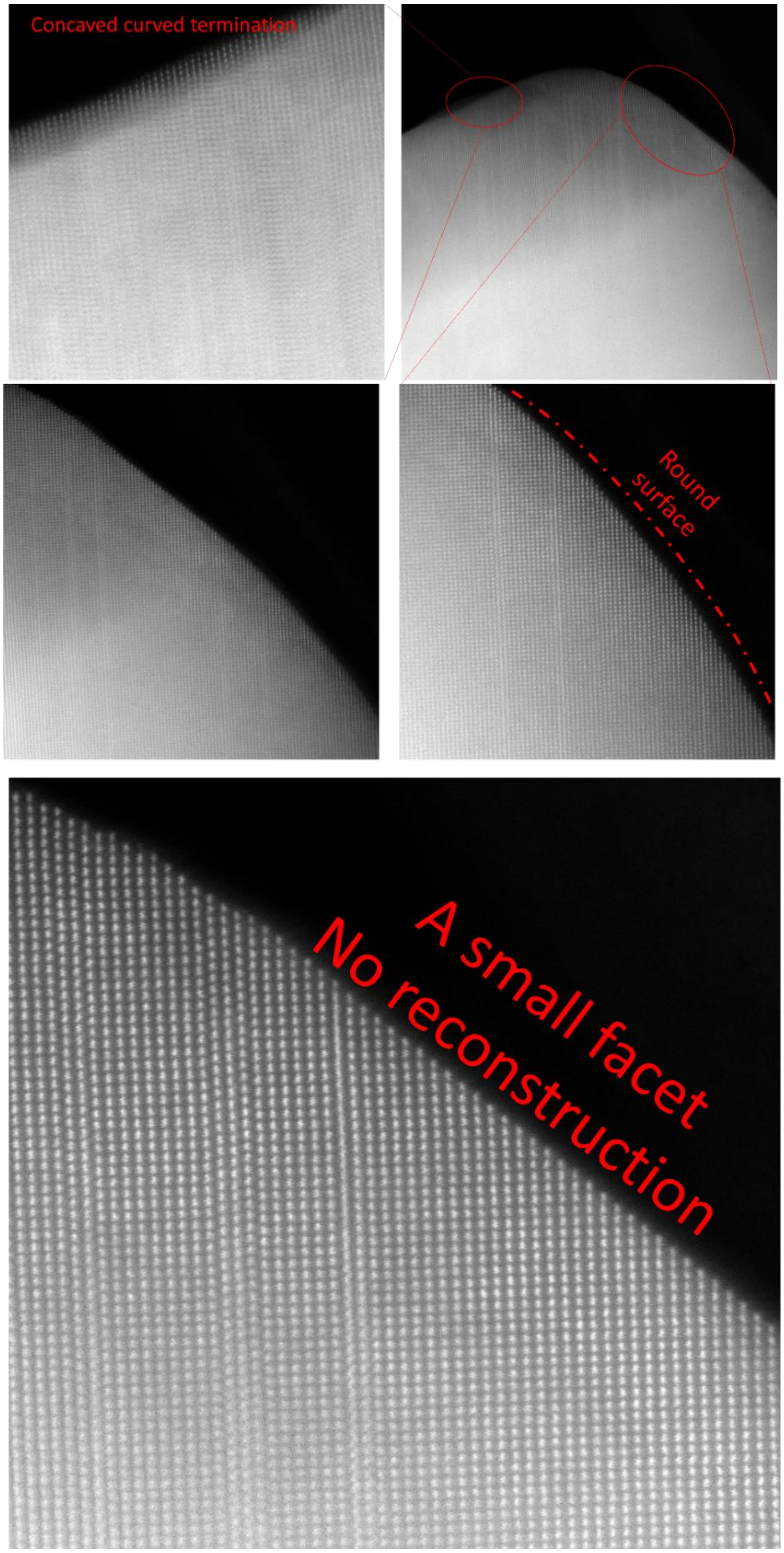

Supplementary Figure 26. Atomic resolution imaging of the termination of a pristine LRMO particle.

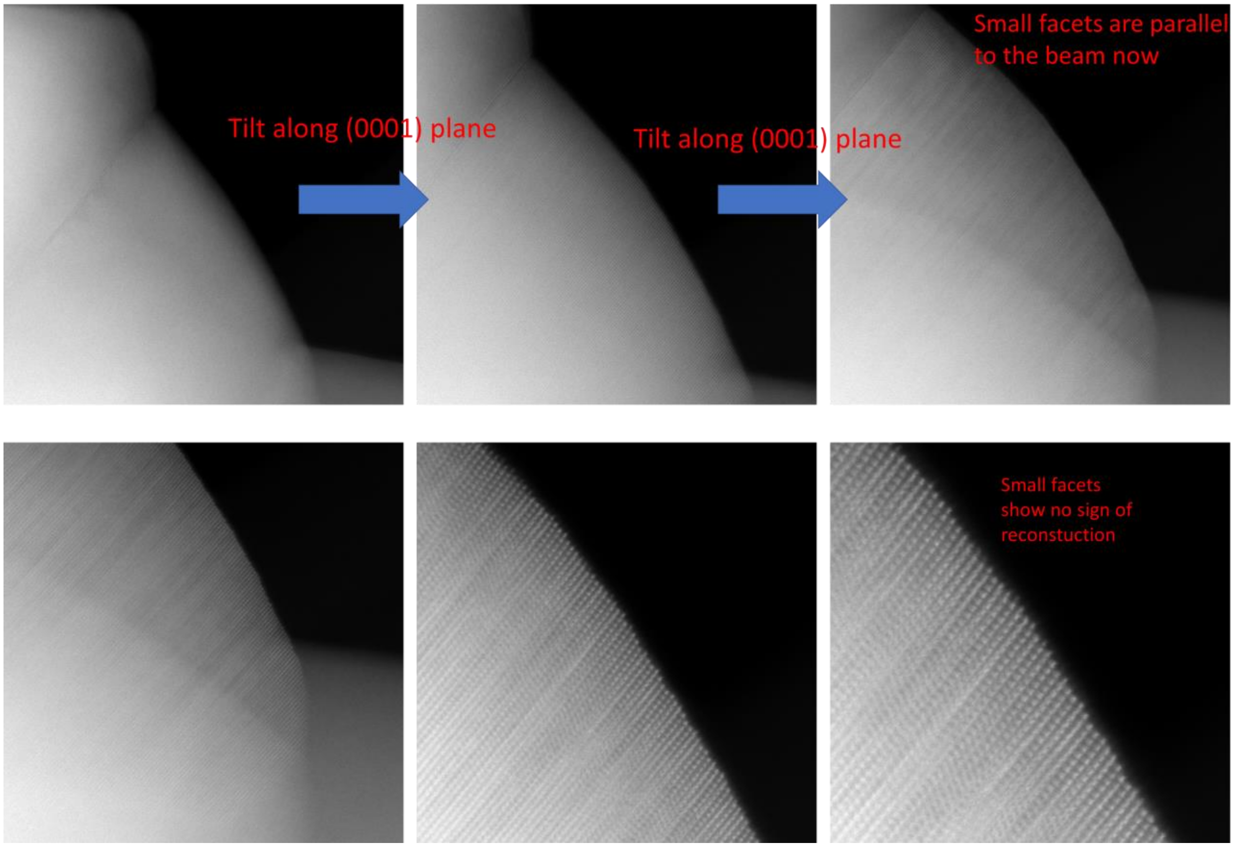

Supplementary Figure 27. Atomic resolution imaging of the termination of a pristine LRMO particle.

Supplementary Tables

Supplementary Table 1. Crystal structure, magnetic configuration and formation energy of transition metal oxides

TMO	Crystal structure	Total magnetization (Bohr mag/cell)		Absolute magnetization (Bohr mag/cell)		Magnetic ordering		Formation energy (eV/formula)					U
		GGA	GGA+U	GGA	GGA+U	GGA	GGA+U	GGA	GGA+U	GGA^{I} [a]	$GGA + U^{I}$	$GGA + U^{II}$ [b]	
						3*d* TMO							
CaO	*Fm*-3*m*	0.0		0.0		NM[c]		-6.415		-7.094			
ScO	*Fm*-3*m*	0.0		0.0		NM		-6.333		-7.013			
TiO	*Fm*-3*m*	0.0		0.01		AFM[d]		-5.137		-5.817			
VO	*Fm*-3*m*	0.0	0.0	4.56	5.75	AFM	AFM	-4.013	-1.977	-4.693	-2.657	-3.539	3.25
CrO	*Fm*-3*m*	0.0	0.0	6.93	7.94	AFM	AFM	-3.050	-1.207	-3.730	-1.887	-2.920	3.7
MnO	*Fm*-3*m*	0.0	0.0	9.03	9.53	AFM	AFM	-3.175	-2.218	-3.855	-2.898	-3.741	3.9
FeO	*Fm*-3*m*	0.0	0.0	7.1	7.6	AFM	AFM	-2.457	-0.021	-3.137	-0.701	-1.563	5.3
CoO	*Fm*-3*m*	0.0	0.0	5.07	5.35	AFM	AFM	-1.976	-0.255	-2.656	-0.935	-1.811	3.32
NiO	*Fm*-3*m*	0.0	0.0	2.97	3.62	AFM	AFM	-1.721	-0.353	-2.401	-1.033	-2.115	6.2
CuO	*Fm*-3*m*	0.0		0.0		NM		-1.333		-2.013			
ZnO	*Fm*-3*m*	0.0		0.0		NM		-3.085		-3.765			
						4*d* TMO							
SrO	*Fm*-3*m*	0.0		0.0		NM		-5.957		-6.637			
YO	*Fm*-3*m*	0.0		0.0		NM		-6.115		-6.795			
ZrO	*Fm*-3*m*	0.0		0.0		NM		-5.123		-5.803			
NbO	*Fm*-3*m*	0.0		0.0		NM		-3.466		-4.146			
MoO	*Fm*-3*m*	0.0		0.0		NM		-2.416		-3.096			
RuO	*Fm*-3*m*	0.0		0.0		NM		-0.488		-1.168			
RhO	*Fm*-3*m*	0.0		0.0		NM		-0.131		-0.811			
PdO	*Fm*-3*m*	0.0		0.0		NM		-0.444		-1.124			
AgO	*Fm*-3*m*	0.0		0.0		NM		-0.457		-1.137			
CdO	*Fm*-3*m*	0.0		0.0		NM		-2.513		-3.193			

[a] superscript denotes shifted O2 energy (correction to O_2)
[b] superscript denotes energy correction according to Jain *et al.* (correction to mixing GGA and GGA+U)
[c] Nonmagnetic
[d] Antiferromagnetic

Supplementary Methods

MnO computational detail. We calculated the formation energy of a series of rock-salt structure MO, as M for all 3*d* and 4*d* transitional metals, except for technetium (Tc) which has radioactivity. To compare the formation energy among different species and eliminate the possible artificial effect of Hubbard U, these calculations were carried out without the Hubbard U correction (see onsite U section below for detailed explanation). For a supercell containing four formulas of MO, a much denser k-point mesh of 6x6x6 were employed for each structure.

MnM_xO, NiM_xO, CoM_xO computational detail. We also considered the solubility of all 3*d*/4*d* transition metals in $T_xM_{1-x}O$ (T = Mn, Ni, Co; M = 3*d*/4*d* species) rock-salt structure. A supercell containing four units of $T_xM_{1-x}O$ with a k-point mesh of 6x6x6 was employed. The enumeration strategy documented by Hart *et al*[4, 5, 6] was used to generate T/M ordering for each composition (as x = 0.25, 0.5, 0.75).

Lithium-rich nickel-titanium-niobium oxide. To test our theoretical calculation, we synthesized lithium-rich nickel-titanium-niobium oxide, namely $Li_{1.21}Ti_{0.5}Ni_{0.22}Nb_{0.07}O_2$ (LNTN), by a solid reaction method. This material features cation disordering and retains a Fm3m space group. The electrolyte was $LiPF_6$ (1 M) in ethylene carbonate and dimethyl carbonate with a volume ratio of 1:1. 2032 coin cells were assembled in an argon-filled glove box. Battery testing was performed on a computer controlled galvanostat (model LandCord 5.8). Cells were charged/discharged at a current density of 100 mA g^{-1}. The electrochemical data is shown in Supplementary Figure 10. Even though this material has significant capacity fading over 50 charge/discharge cycles, no niobium segregation was observed in the near-surface area of the cathode material (see Supplementary Figs. 11, 12, and 13).

Hubbard U. Hubbard *U* correction is very important for predicting the correct band structure but it introduces arbitrary instability for formation energy calculation. G. Ceder *et al* proposed instead to calculate formation enthalpies by mixing GGA and GGA + U empirically (PRB, 2011, 84(4), 045115[7]). Therefore, we internally benchmarked GGA against the method proposed in two papers of Ceder and his coauthors[7, 8], including the energy correction to O_2 molecular and GGA+U correction to TMOs. The results are shown in Supplementary Table 1 and Supplementary Figure 18. Our conclusions are

(1) As Supplementary Figure 18 shows, the magnitude of U value has a vast influence on the formation energy of TMOs. Without a proper U value, it is not possible to get a convincing formation energy of TMOs within DFT+U method.

(2) Apart from FeO and CoO, it is nearly the same trend of formation energy of TMOs among different calculation methods, involving GGA + U^{II}(suggested by Ceder et al. [7]) , ordinary GGA and GGA^{I} (corrections to O_2 [8]).

(3) Since our is to find the TMOs with larger oxygen formation energy, from this perspective, the results of GGA, GGA^{I}, and the empirical GGA/GGA+U hybrid method share the same trend. To stay in the ab initio frame work without having to introduce empirical parameters, we decided to use GGA for our formation energy calculation.

Supplementary References

1. Sparrow, T., Williams, B., Rao, C. & Thomas, J. L3/L2 white-line intensity ratios in the electron energy-loss spectra of 3d transition-metal oxides. *Chem. Phys. Lett.* **108**, 547–550 (1984).

2. Schmid, H. K., & Mader, W. Oxidation states of Mn and Fe in various compound oxide systems. *Micron* **37**, 426–432 (2006).

3. Tan, H., Verbeeck, J., Abakumov, A. & Tendeloo, V. G. Oxidation state and chemical shift investigation in transition metal oxides by EELS. *Ultramicroscopy* **116**, 24-33 (2012).

4. Hart, G. L. & Forcade, R. W. Algorithm for generating derivative structures. *Phys. Rev. B* **77**, 224115 (2008).

5. Hart, G. L. & Forcade, R. W. Generating derivative structures from multilattices: Algorithm and application to hcp alloys. *Phys. Rev. B* **80**, 014120 (2009).

6. Hart, G. L., Nelson, L. J. & Forcade, R. W. Generating derivative structures at a fixed concentration. *Comput. Mater. Sci.* **59**, 101–107 (2012).

7. Jain, A. *et al.* Formation enthalpies by mixing GGA and GGA+U calculations. *Phys. Rev. B* **84**, 045115 (2011).

8. Wang, L., Maxisch, T. & Ceder, G. Oxidation energies of transition metal oxides within the GGA+ U framework. *Phys. Rev. B* **73**, 195107 (2006).
