# Supplementary material for: Anomalous metal segregation in lithium-rich material provides design rules for stable cathode in lithium-ion battery
Source: Nat Commun. 2019 Apr 9;10:1650. doi: 10.1038/s41467-019-09248-0 (PMC6456622; doi:10.1038/s41467-019-09248-0)
Supplement: Supplementary file 2 — Description of Additional Supplementary Files [file 41467_2019_9248_MOESM2_ESM.pdf]

Description of Additional Supplementary Files

File Name: **Supplementary Movie 1.**

Description: 3D visualization of the chemically gradated structure of a pristine LRMO primary particle.

File Name: **Supplementary Movie 2.**

Description: Cross-sectional movie of the 3D tomographic reconstruction of a pristine LRMO primary particle showing that the internal structure of the particle is solid.

File Name: **Supplementary Movie 3.**

Description: 3D visualization of the sub-particle scale chemical inhomogeneity of a LRMO primary particle that underwent 97 cycles.

File Name: **Supplementary Movie 4.**

Description: Cross-sectional movie of the 3D tomographic reconstruction of a LRMO particle after 97 cycles showing that the internal structure of the particle is porous.

File Name: **Supplementary Movie 5.**

Description: Cross-sectional movie of the 3D STEM-EDX tomographic reconstruction of a pristine LRMO particle.

File Name: **Supplementary Movie 6.**

Description: Cross-sectional movie of the 3D STEM-EDX tomographic reconstruction of a LRMO particle after 97 cycles.

File Name: **Supplementary Movie 7.**
Description: Cross-sectional movie of the 3D oxygen distribution of a LRMO particle after 97 cycles.
